# Phytochemical Profiling, In Vitro and In Silico Anti-Microbial and Anti-Cancer Activity Evaluations and Staph GyraseB and *h*-TOP-IIβ Receptor-Docking Studies of Major Constituents of *Zygophyllum coccineum* L. Aqueous-Ethanolic Extract and Its Subsequent Fractions: An Approach to Validate Traditional Phytomedicinal Knowledge

**DOI:** 10.3390/molecules26030577

**Published:** 2021-01-22

**Authors:** Hamdoon A. Mohammed, Riaz A. Khan, Atef A. Abdel-Hafez, Marwa Abdel-Aziz, Eman Ahmed, Shymaa Enany, Sebaey Mahgoub, Osamah Al-Rugaie, Mansour Alsharidah, Mohamed S. A. Aly, Ahmed B. M. Mehany, Mostafa M. Hegazy

**Affiliations:** 1 Department of Medicinal Chemistry and Pharmacognosy, College of Pharmacy, Qassim University, Qassim 51452, Saudi Arabia; a.abdelgalil@qu.edu.sa; 2 Department of Pharmacognosy, Faculty of Pharmacy, Al-Azhar University, Cairo 11371, Egypt; mostafahegazy@azhar.edu.eg; 3 Regional Center for Mycology and Biotechnology (RCMB), Al-Azhar University, Cairo 11371, Egypt; marwaemam.17@azhar.edu.eg; 4 Department of Pharmacology, Faculty of Veterinary Medicine, Suez Canal University, Ismailia 41522, Egypt; eman.abdelnaby@57357.org; 5 Proteomics and Metabolomics Unit, Department of Basic Research, Children’s Cancer Hospital Egypt 57357, Cairo 11441, Egypt; Sebaey.Mahgoub@57357.org; 6 Department of Microbiology and Immunology, Faculty of Pharmacy, Suez Canal University, Ismailia 41522, Egypt; Shymaa_enany@pharm.suez.edu.eg; 7 Department of Physiology, Faculty of Veterinary Medicine, Suez Canal University, Ismailia 41522, Egypt; 8 Department of Basic Medical Sciences, College of Medicine and Medical Sciences, Qassim University, Unaizah, P.O. Box 991, Qassim 51911, Saudi Arabia; o.alrugaie@qu.edu.sa; 9 Department of Physiology, College of Medicine, Qassim University, Qassim 51452, Saudi Arabia; m.alsharidah@qumed.edu.sa; 10 Hospital of the Police Academy, Nasr City, Cairo 11765, Egypt; mohamedshawky1974@yahoo.com; 11 Department of Zoology, Faculty of Science, Al-Azhar University, Cairo 11371, Egypt; abelal_81@yahoo.com

**Keywords:** *Zygophyllum coccineum*, chemical profile, LC-ESI-TOF-MS, anti-biofilm, anti-microbial, anti-cancer, MCF-7, HCT-116, HepG2, human topoisomerase-IIβ, Staph Gyrase B, *Candida albicans*, *Staphylococcus pneumoniae*, *Pseudomonas aeruginosa*, *Escherichia coli*

## Abstract

*Zygophyllum coccineum*, an edible halophytic plant, is part of the traditional medicine chest in the Mediterranean region for symptomatic relief of diabetes, hypertension, wound healing, burns, infections, and rheumatoid arthritis pain. The current study aimed to characterize *Z. coccineum* phytoconstituents, and the evaluations of the anti-microbial-biofilm, and anti-cancers bioactivities of the plant’s mother liquor, i.e., aqueous-ethanolic extract, and its subsequent fractions. The in silico receptors interaction feasibility of *Z. coccineum* major constituents with Staph GyraseB, and human topoisomerase-IIβ (*h*-TOP-IIβ) were conducted to confirm the plant’s anti-microbial and anti-cancer biological activities. Thirty-eight secondary metabolites of flavonoids, stilbene, phenolic acids, alkaloids, and coumarin classes identified by LC-ESI-TOF-MS spectrometric analysis, and tiliroside (kaempferol-3-O-(6′′′′-*p*-coumaroyl)-glucoside, 19.8%), zygophyloside-F (12.78%), zygophyloside-G (9.67%), and isorhamnetin-3-O-glucoside (4.75%) were identified as the major constituents. A superior biofilm obliteration activity established the minimum biofilm eradication concentration (MBEC) for the chloroform fraction at 3.9–15.63 µg/mL, as compared to the positive controls (15.63–31.25 µg/mL) against all the microbial strains that produced the biofilm under study, except the *Aspergillus fumigatus*. The aqueous-ethanolic extract showed cytotoxic effects with IC_50_ values at 3.47, 3.19, and 2.27 µg/mL against MCF-7, HCT-116, and HepG2 cell-lines, respectively, together with the inhibition of *h-*TOP-IIβ with IC_50_ value at 45.05 ng/mL in comparison to its standard referral inhibitor (staurosporine, IC_50_, 135.33 ng/mL). This conclusively established the anti-cancer activity of the aqueous-ethanolic extract that also validated by in silico receptor-binding predicted energy levels and receptor-site docking feasibility of the major constituents of the plant’s extract. The study helped to authenticate some of the traditional phytomedicinal properties of the anti-infectious nature of the plant.

## 1. Introduction

The *Zygophyllum coccineum* is a halophytic plant species with specific ability to survive in the high-salted, marshy, and coastal-land areas. The plant belongs to the Zygophyllaceae family which includes over 200 species [1]. Like any other halophytes, the adeptness of *Z. coccineum* to withstand high salinity is attributed to its ability to produce specific metabolites of primary and secondary nature which works as anti-oxidants, phytoalexins, and chemical and biomechanistic arbitrator to regulate different unfavorable conditions of the environment to help plant handle the physiological, biochemical and pathological effects of the harsh surroundings [2]. Phytochemically, the *Z. coccineum,* and the family Zygophyllaceae are reputed for their presence of quinovic acid glycosides. An earlier investigation on *Z. coccineum* also reported the presence of triterpene-saponins [3,4,5], flavonoids, and sterol glycosides [3,6]. The plant possesses potent anti-oxidant activity and has traditionally been used for diabetes, hypertension, wound-healing, burns, infections, gout, rheumatoid fever, and pain, including its use as an anthelmintic, and diuretic agents [5,7]. Previous investigations have also revealed anti-microbial [8,9] and in vitro cytotoxic effects of *Z. coccineum*, especially against MCF-7 and HeLa cell-lines [8].

The anti-cancer and anti-microbial bioactions of plants of varying climatic zones and environmental conditions, including halophytes, have supposedly been considered as a reservoir of new structural templates to exploit and extricate the active constituents in the worldwide quest for new anti-cancer and anti-microbial agents to combat the evolving resistance for effective chemotherapy [10,11]. Among various modes of anti-microbial actions, the biofilm formation by pathogens causes persistent and recurring infections that are resistant to therapy with conventional treatments, and up to 80% of humans’ microbial infections are biofilm-associated [12]. The fungal biofilm perpetrated infections are also extremely difficult to eradicate owing to the capability of the produced biofilm to protect the fungal pathogens from the innate immune system of the host, and disruption to the obliterating effects of the anti-fungal drugs. Such infections are most frequently caused by the *Candida*, and *Aspergillus* species [13,14]. Several plants, especially of arid and semi-arid zones, have demonstrated effective anti-microbial activity, also working against these two microbial species to control these infections [2]. However, precipitous interest in new plant species having potent anti-microbial activity has increased over time, partly due to varying modalities of the biomechanism of the anti-fungal and anti-microbial agents as well as contagion resistance to existing anti-microbial vis à vis anti-fungal treatments. The roles of phytoconstituents, their structural specifications, extent and outreach, and durability of the anti-microbial actions are other considerations of interest in searching and selecting new plants for bio-screenings for their attendant new and diversified anti-microbial structural templates capable of eradicating the infections in an effective and durable manner. The presence of triterpenes, saponins, flavonoids, and phenolics classes of compounds, including the N-based phytoproducts, and more specifically, the role of alkaloids in anti-microbial activity has lately assumed vital significance [15]. Nonetheless, the relationship of higher contents of flavonoids and phenolics of varied structural templates as part of the plants extracts’ constituents have amply suggested varying roles of the anti-oxidant activity of the plants of the harsh habitat for their bioaction against the microbes. The confirmed antimicrobial potential of plants with higher contents of flavonoids, phenols, and other anti-oxidants are amply reported [16,17]. However, in retrospect, a limited number of references pertaining to the anti-oxidant activity of flavonoids and phenolics with structurally-defined anti-microbial agents are encountered. The examples of quinovic acid, *p*-hydroxybenzoic acid, salicylic acid, and other flavonoids, namely, quercetin, luteolin, and kaempferol derivatives have been shown to possess mild to substantially significant anti-microbial activity [18]. However, the challenge to search better anti-microbials of plant-origins still holds, and efforts to find novel molecular templates is actively pursued in several quarters.

Anti-oxidant compounds, including flavonoids and polyphenols, by virtue of their capability, inhibit the harmful oxidative processes and protection of the body from the damaging effects of free radicals through reducing the reactive oxygen species (ROS) have roles as plausible and potent anti-cancer agents. The interdependence and roles of the ROS and anti-oxidants have been demonstrated, and the relationship of ROS in prognosis and facilitation of various adverse physiological conditions including the damage and malfunctioning of various tissues and organs leading to several diseases and cancerous conditions is well-known [19]. The search for better anti-cancer agents from plant sources has assumed significance, more so, in the light of cancer cells’ resistance to currently available chemotherapeutic agents [20,21] where the existent epidemiological situation has become more predominant. A proportion of more than 80% of cancer-related deaths are associated with single and multi-drug resistance with the prime course of incumbent cancerous conditions relapse and other biochemical and physiological failures which are among a few of the causes to name [22]. Investigations on natural resources, especially plants-derived products, towards new drug discoveries to meet the global health challenges in anti-cancer drug resistance are continued and top priority. In this context, the plants of harsh and drastic environments, including halophytes, have gained momentum due to their survival mechanisms based production of primary and secondary metabolites of varying structural templates, modifications, and chemical functionalities diversifications that are produced to help cope the biochemical, physiological, pathogenic, and xenobiotic challenges of the surrounding ecology of the plant [23]. These products help keep the plant hemostasis, survival instinct, and existence predisposition at expected levels. The quality, nature, ability, and predisposition alters from plant to plant in accordance with the plant physiology and the surroundings, and this has a role to play in the production and role(s) of the plant metabolites of primary and secondary natures [2]. An interest in halophytic plants derived products exhibiting anti-cell-proliferative and apoptosis activity appropriately complements the requirements.

The current investigation aimed to explore some of the vital medicinal properties of the plant, *Z*. *coccineum*, claimed by the traditional herbalist. It also intended to validate the prevalent use of the plant by locals and nomadic folks in various health causes. The study was meant to identify major compounds and the classes of secondary metabolites present in the plant, *Z. coccineum*, and evaluate their biological potential. The biological properties, especially the plant’s anti-microbial and cytotoxic effects, as a source of better anti-microbial and anti-cancer drug/crude drug source for further product development to cope with the drug resistance, were some of the dispositions of the study. To this effect, an LC-MS analysis based profiling of the small molecule secondary metabolites’ presence in the mother liquor aqueous (aq.)-ethanolic extract, and the anti-microbial and anti-cancer activities of the mother liquor aq.-ethanolic extract, and its subsequent fractions against MCF-7, HCT-116, and HepG2 cell-lines were planned and investigated. The anti-microbial and anti-cancer activities were reconfirmed through in vitro binding of the Staph GyraseB and human topoisomerase-IIβ (*h*TOP-IIβ), respectively, with the aq.-ethanolic extract’s major constituents. The anti-cancer and anti-microbial activities were validated through in silico receptor-ligand docking studies to provide an insight into the level of the binding feasibility. The investigation assisted to authenticate some of the traditional phytomedicinal reputations of the plant.

## 2. Results

### 2.1. Total Phenolic and Flavonoid Contents

The total phenolic and flavonoid contents were measured as 24.9 ± 1.7 and 23.84 ± 2.1 mg/gm of the dried extract in the *Z. coccineum* aq.-ethanolic extract as equivalents of gallic acid (GAE) and rutin (RE) per gram of the extract, respectively, though it were anticipated to be much higher to align with the halophytic category specifics of the plant. Other Zygophyllum species contained higher concentrations of phenolic and flavonoid contents, sometimes up to 10× higher, e.g., *Z. album* leaves contained 296 and 216 mg/g of the total phenolic and the total flavonoid contents, respectively [24]. However, previous reports on the *Z. coccineum* total phenolic and flavonoids contents concentration in the plants are consistent with our current results [25].

### 2.2. Identification and Profiling of Z. coccineum Constituents by LC–ESI-TOF–MS

Negative and positive modes of ESI masses were used in the identification of 38 compounds in the *Z. coccineum* herb. The compounds included stilbene derivative, phenolic acids, flavonoids, sterols, alkaloids, and saponins (Table 1) arranged according to their RT (retention time). The MS-DIAL 3.70 open-source software [26] was used in the identification of products while ReSpect positive (2737 records) or ReSpect negative (1573 records) databases were used as the reference database.

**Figure 1:**
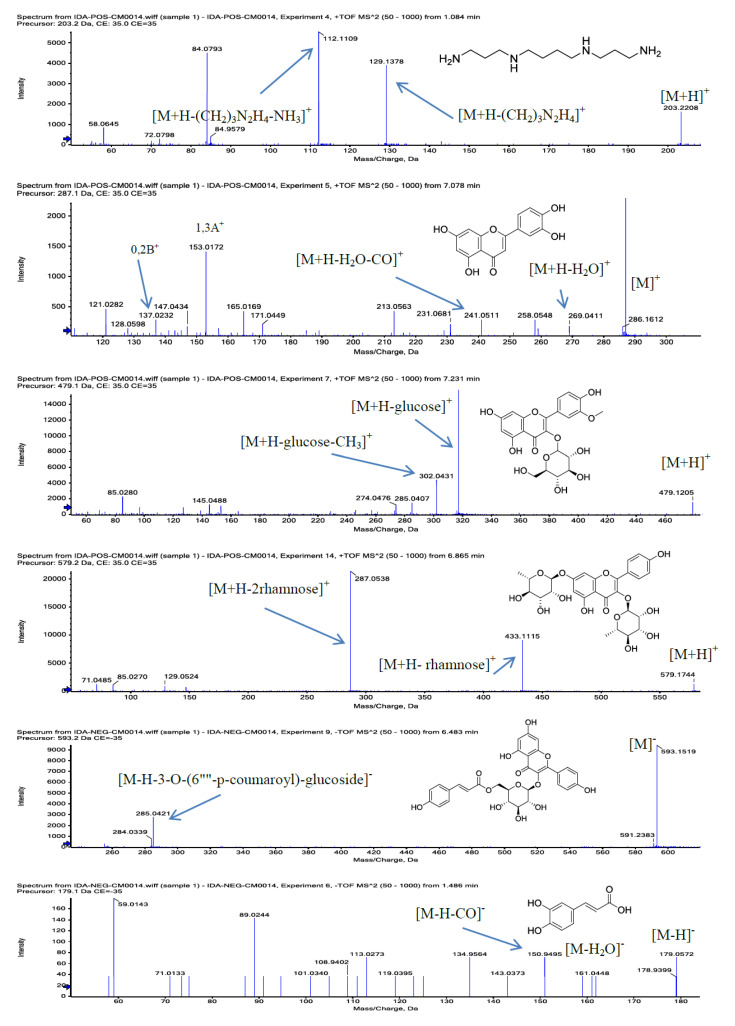

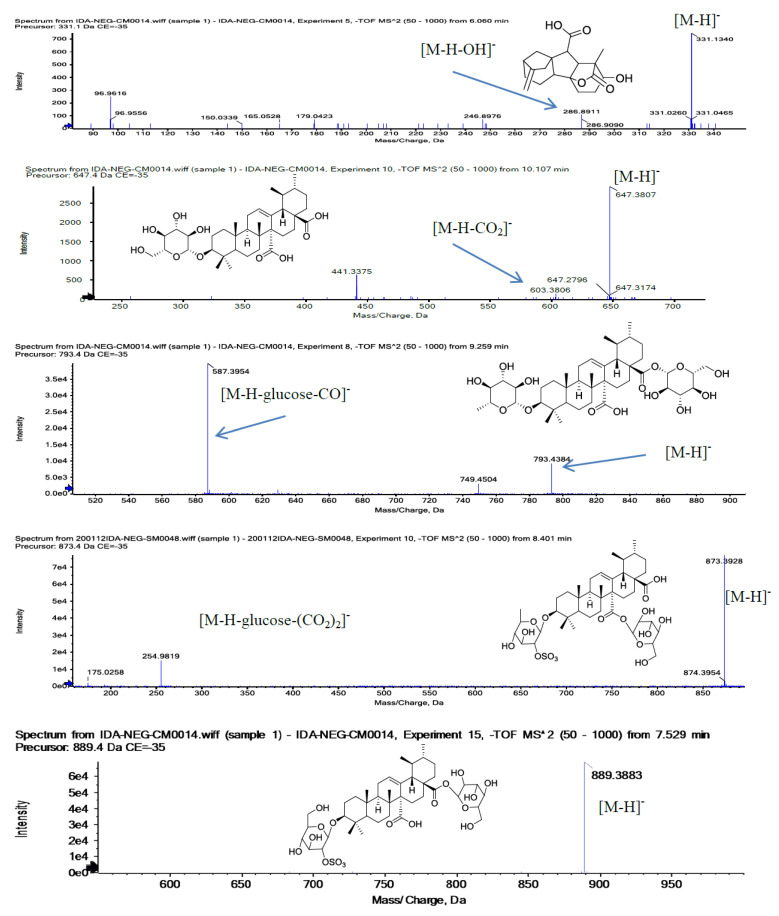
Structures and mass fragments of major identified constituents in *Z. coccineum* aq.-ethanolic extract in both negative and positive modes, from top to bottom: (i). Spermine (entry # **1**), (ii). Luteolin (entry # **27**), (iii). Isorhamnetin-3-O-glucoside (entry # **28**), (iv). Kaempferol 3,7-di-O-α-L-rhamnoside (entry # **24**), (v). Tiliroside (entry # **21**), (vi). Caffeic acid (entry # **4**), (vii). Gibberellin-A4 (entry # **14**), (viii). 3-*O*-[β-D-gluco pyranosyl] quinovic acid (entry # **34**), (ix) 3-*O*-[β-D-Quinovopyranosyl] quinovic acid-28-β-D-glucopyranosyl ester (entry # **32**), (x). Zygophyloside-F (entry # **31**), and (xi). Zygophyloside-G (entry # **29**) in Table 1.

The fragmentation patterns (Figure 1), and previous reports on the phytoconstituents of *Z. coccineum* and other plants of the genus Zygophyllum were referenced in the identification of the compounds. Additionally, the relative percentages of the identified compounds in the plant extracts were calculated based on the total peak area in the chromatogram (Table 1).

The majority of the identified compounds (Table 1) were phenolics and flavonoids in nature, the hallmarks of the salt-stressed plants [27], thereby reflecting the halophytic nature of the plant, and its ability to biosynthesize patterns of phenolics and flavonoids as part of the possible mechanism to overcome oxidative stress produced in response to high-salinity conditions [2]. The eighteen flavonoids and six phenolic compounds (Table 1) were characterized from the aq.-ethanolic extract of the plant with a relative percentage value of 32% of the constituents of the total extract. The eighteen flavonoids listed in Table 1 includes three flavonol aglycones, eight flavonol glycosides, two flavones, and two dihydroxyflavones, in addition to, one flavone glycoside, one isoflavone, and one anthocyanin category of compounds. The mass fragments of A and B-rings, in addition to small molecules fragments, i.e., 18 atomic mass unit (amu) for H_2_O, 28 amu for C=O, and 42 amu for CH_2_C=O were used as structure elucidation markers for the flavonoid aglycones (Table 1, Figure 1) [28]. The sugar/s type in the flavonoid glycosides was confirmed by the MS fragmentation patterns in the respective spectra of these compounds, i.e., subtraction of 162 amu and 146 amu revealed elimination of glucose and rhamnose units from the corresponding glycone which was observed in the fragmentation patterns of the quercetin-4′-O-glucopyranoside (**13**), and rutin (**19**), respectively. The chemotaxonomic marker of the genus Zygophyllum, i.e., isorhamnetin-3-O-rutinoside (**25**) [3,29,30,31,32] was identified in the *Z. coccineum* extract with a negative precursor, *m*/*z* 623.2877 [M-H]^−^ and the relative percentage value at 0.076%. The flavonoids were represented at 30.35% of the total LC-MS chromatogram constituents while two-third of this percentage were calculated to be tiliroside (kaempferol-3-O-(6′′′′-*p*-coumaroyl)-glucoside) (**21**) (Figure 1), as the major flavonoid glycoside content in the plant’s mother liquor, aq.-ethanolic extract. The compounds **19**, **20**, **25**, and **28** have also been previously identified in plant extracts of genus Zygophyllum [30,33]. However, all other identified flavonoids were recorded for the first time from the genus *Zygophyllum* and the species, *Z. coccineum*.

Six phenolic acids were also identified (Table 1). The structures of these acids were elucidated through their molecular ion mass peaks and their fragmentation patterns’ characteristics, i.e., the presence of caffeic acid was confirmed by the observation of molecular ion peak at *m*/*z* 180.0419 [M]^+^, base peak at *m*/*z* 179.0386 [M − H]^+^, and fragments ion peaks at *m*/*z* 135, 150 and 161 for [M-CO_2_H]^+^, [M-COH+H]^+^, and [M − H_2_O + H]^+^, respectively, in the positive mode, while the observations of the mass peaks at *m*/*z* 179 [M − H]^−^, 161 [M − H − H_2_O]^−^, and 150 [M − H − CO]^−^ in the negative mode mass confirmed the caffeic acid structure (Figure 1). Three of the phenolic acids were also previously identified from the genus Zygophyllum, i.e., caffeic acid (**4**), *p*-coumaric acid (**6**), and sinapic acid (**22**) [34,35]. However, this is the first report of these phenolic acids’ presence in the *Z. coccineum* species. Besides, 2-hydroxyphenyl acetic acid (**3**), salicylic acid (**5**), and *p*-hydroxybenzoic acid (**9**) were also identified for the first time from the genus Zygophyllum and the plant *Z. coccineum*.

The triterpenoid saponins have also been reported from *Z. coccineum* and the other species of the genus Zygophyllum [4,5,6,29,36,37,38]. Additionally, the quinovic acid derivatives were considered as the major secondary metabolites in *Z. coccineum* [4,6], and five quinovic acid-based triterpenoid saponins (compounds entered at serial #**23**, **29**, **31**, **32**, and **34** in Table 1) were identified with a relative percentage value of 27.12% of the total plant extract’s constituents. All the identified triterpenoid saponins were also recorded in previous studies for *Z. coccineum* [5,6,36]. Moreover, these types of saponins (quinovic acid-based triterpenoid saponins) were reported from the genus Zygophyllum and might be considered a chemical marker for the genus. The presence of 3-*O*-[β-D-quinovopyranosyl] quinovic acid-28-β-D-glucopyranosyl ester, zygophyloside-G, zygophyloside-F, and zygophyloside-K were also previously identified in *Z. album*, *Z. decumbens*, and *Z. dumosum* plants [29,37,39].

The LC–ESI-TOF–MS analysis also led to identifying other compounds from the miscellaneous category from the aq.-ethanolic extract of the *Z. coccineum*. These compounds included three alkaloids, i.e., spermine (**1**), spermidine (**2**), and kynurenic acid (**8**), together with a stilbene glycosides, i.e., isorhapontin (**7**); one sesquiterpene identified as abscisic acid (**10**); a tetracyclic-diterpene acid, i.e., gibberellin-A4 (**14**), two aldehydes, i.e., syringaldehyde (**16**), and cinnamaldehyde (**30**), and one coumarin which was identified as 7-acetoxy-4-methyl coumarin (**17**). These compounds were recognized for the first time from the plant, *Z. coccineum,* and the genus Zygophyllum. These products were identified according to data obtained from their molecular ion masses, mass fragments in comparison with the literature mass values [39,40,41,42,43,44,45], and ReSpect databases.

### 2.3. Anti-Microbial Biofilm Activity of Z. coccineum aq.-Ethanolic Extract and Subsequent Fractions

The anti-microbial activity of *Z. coccineum* has been established in previous reports [8,9]. However, the plant’s effects against microbial biofilms, which indicate the plant’s potential for resolving one of the microbial resistance mechanisms are being reported in this study for the first time. Moreover, the diversity of secondary metabolites present in *Z. coccineum*, e.g., alkaloids, phenolic acids, flavonoids, saponins, and phenolic aldehydes (Table 1) prompted us to investigate the plant’s potential as an anti-microbial-biofilm agent. The identified constituents of the plant were good indicators of the anti-microbial activity of the plant, i.e., some of the currently encountered plant phenolic acids have been used as preservatives with powerful anti-microbial effects, e.g., *p*-hydroxybenzoic acid, salicylic acid, and their esters for pharmaceuticals, and cosmetic preparations [65,66].

Table 2 summarized all the anti-microbial activity results that include the MICs, MBICs, and MBECs for the aq.-ethanolic extract and its subsequent fractions, i.e., chloroform, ethyl acetate, and *n*-butanol. The results in Table 2 revealed that the chloroform fraction was the best performer among all the other fractions against all the microbial strains with MIC values ranging from 0.49 to 1.95 µg/mL, MBIC values ranged between 0.24 to 0.12 µg/mL, and MBEC values were between 3.9 to 15.63 µg/mL. Moreover, the chloroform extract eradicated all microbial strains at concentrations lower than that recorded for the positive controls except for the *Aspergillus fumigatus* that was obliterated with chloroform fraction and amphotericin B at a similar concentration (15.63 µg/mL). The potential anti-microbial activity was also exhibited by the ethyl acetate fraction, which eradicated the microbial biofilm of the tested microorganisms at a concentration range of 31.25 to 62.5 µg/mL with similarities with the positive controls. The results also confirmed the moderate anti-microbial activity for the aq.-ethanolic extract while the *n*-butanol fraction was the weakest in its anti-microbial actions. The potential anti-microbial effects of chloroform and ethyl acetate fractions could be related to the presence of flavonoid glycosides and aglycones, phenolic aldehydes and acids, stilbene, and coumarin constituents of the plant which were extracted by the moderately polar solvents in these fractions.

### 2.4. Anti-Cancer Activity of Z. coccineum: aq.-Ethanolic Extract, Chloroform, Ethyl Acetate, and n-Butanol Fractions

The anti-cancer effects of *Z. coccineum* aq.-ethanolic extract and its subsequent fractions were evaluated against all three-cancer cell lines, i.e., MCF-7 breast cancer, HGT-116 colon cancer, and HepG2 hepatic cancer. The results summarized in Table 3 showed low IC_50_ values for the aq.- ethanolic extract at 3.47, 3.19, and 2.27 µg/mL against MCF-7, HCT-116, and HepG2 cell lines, respectively, indicating the significant anti-cancer activity of the plant. Among all the fractions derived from the aq.-ethanolic mother liquor, the chloroform fraction was the most active with IC_50_ values at 5.78, 4.12, and 3.17 µg/mL against MCF-7, HCT-116, and HepG2 cell lines, respectively. The comparatively higher cytotoxic activity of the aq.-ethanolic extract over its other fractions is indicative of either the synergistic effects of *Z. coccineum* constituents or comparatively higher concentration of the active constituent in the aq.-ethanolic mother liquor than the fractions derived from it. The presence of a minor quantity of the active constituent exhibiting anti-cancer activity in the fractions is also one of the possibilities. However, the weaker anti-cancer activity of the chloroform, ethyl acetate, and *n*-butanol fractions with the presence of multiple constituents that are responsible for exhibiting the bioactivity against the MCF-7, HCT-116, and HepG2 cell-lines, as compared to the mother liquor, was also suggestive of the presence of lesser concentrations of the active hydrophilic constituent(s) in all these fractions. These fractions were derived from the mother liquor, which is rich in hydrophilic products. Nonetheless, the comparatively higher anti-cancer activity exhibited by the mother liquor, i.e., aq.-ethanolic extract, containing all the constituents in their natural ratio of abundance in the plant, expectedly showed higher anti-cancer activity against these cell-lines, MCF-7, HCT-116, and HepG2. This is an indication of the activity of a single active compound or more probably, the synergistic action of the multiple active constituents of the hydrophilic nature present in the aq.-ethanolic extract. The hydrophilic products mainly containing the flavonoids, their glycosides, and phenolics in the aq.-ethanolic extract of the plant corroborated the anti-oxidant potential of these constituents and their roles in the anti-cancer activity of the extract. A further investigation into the in vitro tests of bioactivity was charted and the topoisomerase-IIβ was the target for assaying the anti-cancer activity of the compounds owing to the essential role of *h-*TOP-IIβ in the proliferation of cells and its indispensable functions in the mitosis stage of cell divisions. The *h-*TOP-IIβ is considered an essential enzyme in cell proliferation studies [67]. The topoisomerase-IIβ inhibition assay was used to interrelate the underlying mechanisms of the cytotoxic effects of the aq.-ethanolic extract. The IC_50_ values at 45.05 ng/mL of the aq.-ethanolic extract (Table 3) compared to the IC_50_ value of the standard topoisomerase-IIβ inhibitor; staurosporine indicated the strongest activity of the plant material in the aq.-ethanolic extract which was observed at being three-folds higher in anti-proliferative action in comparison to the staurosporine. That was also indicative of the cytotoxic mechanism of the extract, probably following the similar biochemical inputs and pathways. Moreover, the in silico receptor-binding and docking feasibility evaluations at the *h-*TOP-IIβ (3QX3) receptor predicted lesser affinity of the aq.-ethanolic extract’s encountered major constituents (Table 4) in comparison with the standard drug, etoposide. However, the summed-up effects of these lesser receptor-affinity-predicted constituents, namely, isorhamnetin-3-O-glucoside, kaempferol 3,7-di-O-α-L-rhamnoside, luteolin, and spermine (all hydrophilic and polar natured compounds) in exhibiting the anti-cancer biological activity cannot be ruled out.

### 2.5. Docking Simulations of the Major Constituents as Anti-Cancer and Anti-Microbial Agents

**Figure 2:**
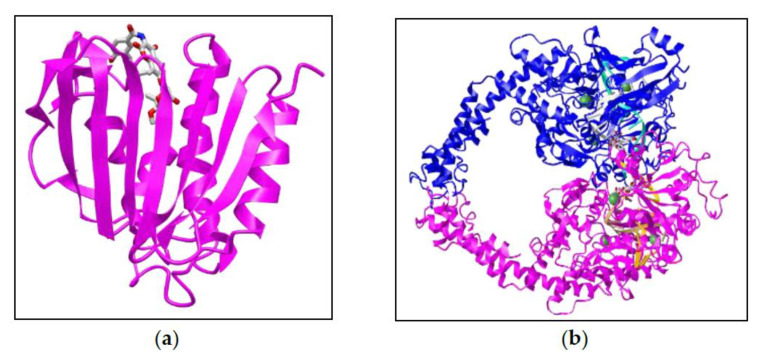
The XRD crystal structures of (**a**) Staph GyraseB (4URO) in complexation with novobiocin (sourced on 5th January 2021 from https://www.ncbi.nlm.nih.gov/Structure/pdb/4URO, 2.59 Å resolution), and (**b**) *h*-TOP-IIβ (Topoisomerase-IIβ/3QX3) in complexation with DNA-etoposide (sourced on 5th January 2021 from https://www.ncbi.nlm.nih.gov/Structure/pdb/3QX3, 2.16 Å resolution).

The molecular docking simulation of the target compounds number **1**, **4**, **14**, **21**, **24**, **27**, **28**, **31**, and **32** (entry #, Table 1), were chosen on the structural category of the compounds, i.e., alkaloid, phenolic acid, phytoalexin, and flavonoid derivative. The docking simulations were performed by Molecular Operating Environment (MOE) *version* 2019.0101, using Staph GyraseB 24 kDa (PDB Id: 4URO), and *h-*TOP-IIβ (PDB Id: 3QX3) (Figure 2a,b), in comparison with the known anti-microbial standard compound, novobiocin, and anti-cancer standard molecule, etoposide as templates to rationalize the anti-microbial, and anti-cancer activity of *Z. coccineum* aq.-ethanolic extracts’ identified compounds. The exercise enabled the prediction of geometric disposition, stereo-orientation, and interaction levels of these compounds at both the receptor’s active sites, the Staph GyraseB and the hTOP-IIβ. The binding free energy, ΔG, values in kcal/mole were recorded as a scoring function, which reflected the output of the docking simulations, and that was proportional to the sum of the Gaussian R_1_R_2_ exp (0.5d^2^), where R_1_ and R_2_ are the radii of the atoms in Angstrom (Å), and d is the distance between the pair in Å (ASE), a linear combination of values (S, ASE, Econf) where Econf is the estimated self-energy of the ligand in kcal/mole (E).

The docking simulations revealed that tiliroside (**21**), kaempferol-3,7-di-O-α-L-rhamnoside (**24**), luteolin (**27**), isorhamnetin-3-*O*-glucoside (**28**) as the flavonoid constituents, and zygophyloside-F (**31**), 3-O-[β-D-quinovopyranosyl] quinovic acid-28-β-D-glucopyranosyl ester (**32**) that comprise 43.76% (*w*/*w*) of the total identified compounds (Table 1), inhibited the *h-*TOP-IIβ enzyme, and Staph GyraseB at different potency as compared to those of a novobiocin standard (ΔG −6.72) the antibiotic, and the anti-cancer agent etoposide (ΔG −5.94), respectively (Table 4). Tiliroside, kaempferol-3,7-di-*O*-α-L-rhamnoside), and isorhamnetin-3-O-glucoside (26.17%, *w*/*w*, composition) showed the near best ΔG values of −6.47, −6.46, and −6.38 kcal/mole, respectively, in relation to the Staph GyraseB docking energy of the standard anti-microbial ligand, novobiocin, at −6.72 kcal/mole, of which kaempferol-3,7-di-*O*-α-L-rhamnoside (**24**) was the nearest match. On the other hand, isorhamnetin-3-*O*-glucoside (**28**), kaempferol 3,7-di-*O*-α-L-rhamnoside (**24**), luteolin (**27**) as flavonoids (7.85%, *w*/*w*, composition), and spermine (**1,** 0.945%, *w*/*w*, composition) as the alkaloidal constituent demonstrated the best ΔG values at −5.75, −5.65, −5.08, and −5.10 kcal/mole, respectively, relative to the active site binding of etoposide (−5.94 kcal/mole) at *h-*TOP-IIβ receptor-site (Table 4). The lower abundance of isorhamnetin-3-O-glucoside (**28**), kaempferol-3,7-di-O-α-L-rhamnoside (**24**), and luteolin (**27**) constituents in the aq.-ethanolic extract perhaps are indicated as significant in vitro anti-cancer activity of the extract in comparison to the TOP-IIβ inhibitory action (Table 3).

**Figure 3:**
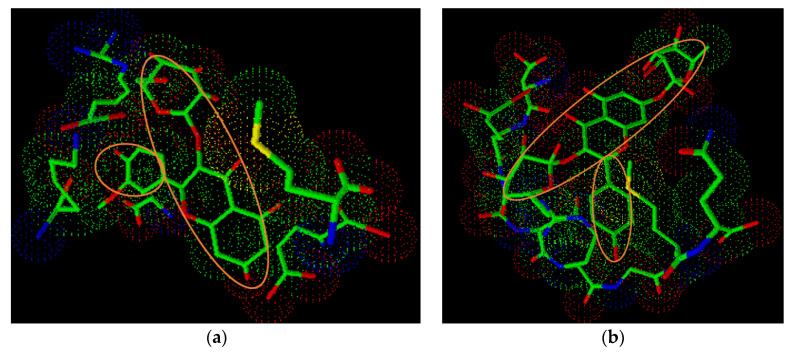
The protein-bound poses of the highest-scoring complexes involving (**a**) isorhamnetin-3-*O*-glucoside, and (**b**) kaempferol 3,7-di-*O*-α-l-rhamnoside (structures in the circles) at the receptor-sites of the 3QX3, and 4URO proteins, respectively.

The binding domains identifying the respective amino acids that are participating in the ligand bindings at the receptor proteins with the *Z. coccineum* major constituents are depicted (Supplementary Information, Table S1). The on-site receptor-protein and ligand-binding domains (Figure 3a,b) for the isorhamnetin-3-O-glucoside within the 3QX3 (*h*-TOP-IIβ) anti-cancer receptor, and kaempferol-3,7-di-O-α-L-rhamnoside binding interaction spaces with the 4URO (Staph GyraseB) anti-microbial substrate protein are represented in analogy to the etoposide and the novobiocin bindings. The ligand-protein binding complexes of the compounds were best fit within the receptor-binding cavity in comparison with the standard ligands. The 4URO (Staph GyraseB) receptor binding of the anti-microbial agent followed the dual-arm, in a nearly U-shaped binding domain. The deep penetration into the binding site pocket and polar interactions of the sugar moiety of the compound with the surface located receptor sites were also observed.

The amino acids sequence Asp^561^Glu^477^Gly^504^Lys^505^Asp^557^Arg^503^Gly^478^Met^782^ flanked by participating H_2_O molecule in hydrogen binding role and complexation with magnesium ion (Mg^++^), found in the interspace between amino acids, Asp^557^ and Arg^503^, and the outlying amino acids His^775^, Leu ^502^, and Gln^778^ contributed to creating the binding cavity domain for the anti-cancer etoposide and the isorhamnetin-3-*O*-glucoside, the nearest scoring *Z. coccineum* compound, as the anti-cancer constituent. The compound binds towards the N-terminal end of the receptor (Figure 3a). The flavonoid rings A, B, and C sits within the receptor site and the sugar ring is involved in the hydrophilic interaction.

The amino acids sequence, Gln^91^Asp^89^Glu^58^Ile^86^Arg^84^Asn^54^Pro^87^, and the other residues, Glu^92^Ile^90^Ile^102^ and ACE124, provided the necessary ratification and support for the ligand-protein binding of the novobiocin and kaempferol-3,7-di-*O*-α-L-rhamnoside at the 4URO receptor cavity (Figure 3b). The orientation of these compounds at the receptors, *h*TOP-IIβ, and Staph GyraseB, offered insights into the potent inhibitory activity of the most abundant products in *Z. coccineum* extract. Based on the binding preferences of different compounds, a receptor binding-site based interactive pharmacophore model, consisting of the on-site amino acids and the generated cavity, was proposed. Based on the comparable energy requirements and the geometric disposition of the incoming ligand in parallel with the standard referral drug ligands, the proposed model can be employed for future activity predictions of other compounds, provided that the cross-resistance to other gyrases are not known. Interestingly, the differential anti-cancer activity of the plant’s aq.-ethanolic extract and its subsequent fractions, namely, ethyl acetate, chloroform, and *n*-butanol, against the cell-lines, i.e., MCF-7, HCT-116, and HepG2, cannot be ruled as synergistic in action owing to the presence of several constituents in these fractions, and the extract. Of the identified compounds (Table 1), a number of these components have exhibited significant levels of closely matching binding energies as compared to the standard ligand. These plant’s products found in the aq.-ethanolic extract and the fractions have also exhibited feasible binding preferences including the geometric dispositions in comparison to the target ligand molecule with the anti-cancer protein (Supplementary File). The binding energy requirements and receptor docking studies indicated probable synergy of the products in exhibiting the strong anti-cancer bioactions of the plant’s constituents present in the aq.-ethanolic extract. Nonetheless, the levels of bioactivity variations, ostensibly, are attributable to various structural differentiations allied to molecular characteristics of the plant’s constituents with their specific ratio of presence in the extract and the fractions. The apparent anti-microbial activity biomechanistics can also not be ruled out of the synergy of the plant’s constituents.

## 3. Materials and Methods

### 3.1. Chemicals, Materials, and Reagents

HPLC grade acetonitrile and methanol were purchased from Thermo-Fisher Scientific (Waltham, MA, USA). Formic acid 98%, ammonium hydroxide, and ammonium formate were purchased from Sigma-Aldrich Co. (St. Louis, MO, USA), XTT [2,3-bis(2-methoxy-4-nitro-5-sulfophenyl)-2*H*-tetrazolium-5-carboxanilide] (Sigma), menadione (Sigma), and all other chemicals were of analytical grades, and purchased from Sigma Aldrich. The cancer cell lines and microbial strains were obtained from the American Type Culture Collection (ATCC, 10801 University Boulevard Manassas, VA 20110 USA). RPMI-1640 medium, Sulforhodamine B (SRB), and dimethyl sulfoxide (DMSO) were purchased from Sigma. Fetal bovine serum (FBS) was obtained from (Gibco, Paisley, UK).

### 3.2. Plant Materials and Extraction Procedure

The plant was collected in 2019 from the high salted arid areas in the Qassim region of central Saudi Arabia and identified by the institutional plant taxonomist at the Qassim University as *Zygophyllum coccineum* L. The plant, as the whole herb, was dried for three weeks in shade and reduced to a coarse powder by mixer-grinder. Approximately, 1 kg of the dried plant material was extracted from aq.-ethanol 70% (5 L, 24 h, three successive extractions). The combined aq. ethanolic extract was evaporated under a vacuum below 40 °C to dryness. The dried aq.-ethanolic extract was then suspended in 0.5 L distilled water and fractionated three times with chloroform, ethyl acetate, and *n*-butanol. Each combined fraction was dried under vacuum <40 °C and dried fractions were stored at −20 °C.

### 3.3. Total Phenolic Contents

The phenolic constituents of *Z. coccineum* aq.-ethanolic extract were calculated as gallic acid equivalent per gram of the dried plant extracts [68]. Mixtures composed of 10 µL of aq.-ethanolic extract (5 mg/mL in methanol), 100 µL of 1:10 diluted Folin Ciocalteu reagent, and 80 µL of 1 M sodium carbonate solution was thoroughly mixed and incubated in dark for 15 min, the developed color was measured spectrophotometrically at 630 nm by the FluoStar Omega microplate reader (BMG LABTECH, Ortenberg, Germany). The standard calibration curve was prepared in the same manner using gallic acid at a concentration range of 500–7.8 µg/mL. The total phenolic contents of the extracts were estimated from the linear regression equation, y = 0.002x − 0.014, R² = 0.998. Triplicate measurement was conducted and the mean with standard deviation was calculated.

### 3.4. Total Flavonoid Contents

The method was proceeded using a microplate assay [69]. A mixture of 100 µL of distilled water and 10 µL of sodium nitrite (NaNO_2_, 50 gm/L), 25 µL of the aq.-ethanolic extract (5 mg/mL in methanol) was mixed and allowed to stand for 5 min before addition of 15 µL of the aluminum chloride solution (AlCl_3_, 10% in ethanol). After 6 min of standing, 50 µL of sodium hydroxide (NaOH, 1 M) and 50 µL of water was added to the mixture. The changed color was measured at 510 nm using FluoStar Omega microplate reader after 30 min shaking. A standard calibration curve was prepared in a similar manner using rutin (1000–50 µg/mL). The process was performed in triplicate and the mean total flavonoid was calculated from the regression equation of the curve, y = 0.001x + 0.013, R² = 0.994.

### 3.5. LC-ESI-TOF-MS Analysis of the aq.-Ethanolic Extract

#### 3.5.1. Sample Preparation

A stock solution of the extract was prepared from 50 mg of the lyophilized aq.-ethanolic extract dissolved in 1000 µL of the solvent mixture composed of water: methanol: acetonitrile (H_2_O:MeOH: ACN) in a ratio of 2:1:1. Complete solubility of stock solution was obtained by sample vortexed and ultra-sonication at 30 kHz for 10 min. An aliquot, 20 µL of the stock solution was again diluted with 1000 µL of the H_2_O: MeOH: ACN (2:1:1) and centrifuged at 10,000 rpm for 5 min, and 10 µL (1 µg/mL) was used for injection. The LC-MS analysis was also performed for blank and quality control samples/internal standard (IS) for confidence in the experiment. The sample was injected in both positive and negative modes.

#### 3.5.2. Instruments and Acquisition Method

Small molecules were separated on an ExionLC system (AB Sciex, Framingham, MA, USA) connected with an autosampler system, an in-line filter disks pre-column (0.5 µm × 3.0 mm, Phenomenex, Torrance, CA, USA), and an Xbridge C18 (3.5 µm, 2.1 × 50 mm) column (Waters Corporation, Milford, MA, USA) was maintained at 40 °C, and at a flow rate of 300 μL/min was utilized. The mobile phase consisted of solution (A): 5 mM ammonium formate in 1% methanol, pH adjusted to 3.0 by using formic acid, and solution (B): consisting of acetonitrile (100%) for the positive mode. The negative mode solution (C): consisted of 5 mM ammonium formate in 1% methanol, adjusted to pH 8 using sodium hydroxide. The gradient elution was performed with the following program: 0–20 min, 10% B; 21–25 min, 90% B; 25.01–28 min, 10% B; and then 90% B for equilibration of the column.

The mass spectrometry (MS) was performed on a Triple TOF 5600+ system equipped with a Duo-Spray source operating in the ESI mode (AB SCIEX, Concord, ON, Canada). The sprayer capillary and declustering potential voltages were 4500 and 80 eV in the positive mode, and −4500 and −80 V in the negative mode. The source temperature was set at 600 °C, the curtain gas was 25 psi, and gas 1 and gas 2 were 40 psi. The collision energy 35 V (positive mode) and −35 V (negative mode) with CE spreading 20 V and the ion tolerance for 10 ppm were used. The TripleTOF5600+ was operated using an information-dependent acquisition (IDA) protocol. Batches for MS and MS/MS data collection were created using Analyst-TF 1.7.1. The IDA method was used to collect full-scan MS and MS/MS information simultaneously. The method consisted of high-resolution survey spectra from 50 to 1100 *m*/*z* and the mass spectrometer was operated in a pattern where a 50-ms survey scan was detected. Subsequently, the top 15 intense ions were selected for acquiring MS/MS fragmentation spectra after each scan [70].

#### 3.5.3. LC-MS Data Processing

MS-DIAL 3.70 open-source software [26] was used for non-targeting, small molecule comprehensive analysis of the sample. According to the acquisition mode, ReSpect positive (2737 records) or ReSpect negative (1573 records) databases were used as reference databases. The search parameters were set as MS1 and MS2 mass tolerance: 0.01 Da and 0.05 Da for data collection, for peak detection; minimum peak height: 100 amplitude, mass slice width: 0.05 Da, smoothing level: 2 scans, minimum peak width: 6 scans, for identification MS1 and MS2 tolerance: 0.2Da/each, for alignment; retention time tolerance: 0.05 min and MS1 tolerance: 0.25 Da. The MS-DIAL output was used to run again on PeakView 2.2 with the MasterView 1.1 package (AB SCIEX) for feature (peaks) confirmation from Total Ion Chromatogram (TIC) based on the criteria; aligned features having Signal-to-Noise ratio greater than 5 and intensities of the sample: blank greater than 5.

### 3.6. Anti-Microbial and Anti-Biofilm Assays

#### 3.6.1. Microorganisms

Biofilm-forming microorganisms were used in the study, including fungi *Aspergillus fumigatus* ATCC 13073, and *Candida albicans* ATCC 10231; Gram-positive bacteria: *Streptococcus pneumoniae* ATCC 6303, *Staphylococcus aureus* ATCC 29213; and Gram-negative bacteria: *Pseudomonas aeruginosa* ATCC 27853, and *Escherichia coli* ATCC 25922.

#### 3.6.2. Determination of Minimum Inhibitory Concentration (MIC)

Anti-microbial activity of aq.-ethanolic mother liquor extract and its subsequent fractions against planktonic tested microorganisms were assessed using the broth dilution method [71]. Overnight cultures of tested microorganisms were grown in brain heart infusion (BHI) broth at mid-logarithmic phase and diluted to 2 × 10^6^ CFU/mL. In polypropylene 96 wells-plate 50 μL of bacteria culture were mixed with 50 μL of the extracts (final concentration two folds’ serial dilutions (0–1000 μg/mL). Plates were incubated at 37 °C for 24 h for bacterial and unicellular fungal strains, and 48 h for the filamentous fungus. The viability of the planktonic tested microorganisms was determined using the XTT assay. The MIC was defined as the lowest concentration of the extracts which inhibited the microbial growths.

#### 3.6.3. Anti-Biofilm Formation Assay

To evaluate the effect of aq.-ethanolic extracts and fractions on the first phase of biofilm formation (attachment), 1 × 10^6^ CFU/mL tested microorganisms were added to 96 wells RBMI plates in two-fold serial dilutions of 0–1000 μg/mL of the extracts and fractions. Plates were incubated for 24 h after which the medium was aspirated gently and the wells of the plates were washed three times with 150 μL phosphate-buffer saline (PBS) to remove unattached cells. The viability of microorganisms in the biofilm was determined using the XTT assay. Minimum biofilm inhibitory concentration (MBIC) was defined as the lowest concentration of the extracts which prevents biofilm formation.

#### 3.6.4. Eradication of Pre-Formed Biofilms Assay

Biofilms were prepared for 24 h concerning bacteria and *Candida albicans* and for 48 h for filamentous fungi in a 96-well tissue culture microtiter-plate containing RBMI media, washed three times with 150 μL PBS. Extracts and fractions in two-fold serial dilutions of 0–1000 μg/mL were added to every well, and the plates were incubated for 24 h at 37 °C. After incubation, the plates were washed three times with 150 μL phosphate-buffer saline (PBS) to remove unattached cells and left to dry. The viability of microorganisms in the pre-formed biofilm was determined using the XTT assay. Minimum biofilm eradication concentration (MBEC) was defined as the lowest concentration of the extracts which eradicated the pre-formed biofilm.

#### 3.6.5. The XTT- Reduction Assay

The viability of the cells was detected using the XTT reduction assay. XTT was prepared in a saturated solution, 0.5 g/liter in Ringer’s lactate. The solution was filtered, sterilized through a 0.22-mm-pore-size filter, aliquoted, and stored at −70 °C. Before each assay, an aliquot of stock XTT was thawed, and menadione (10 mM in acetone) was added to a final concentration of 1 mM. A 100 μL aliquot of the XTT-menadione solution was then added to each well and the control wells (for the measurement of background XTT-reduction levels). The plates were incubated in the dark for 2 h at 37 °C. The activity of the microbial mitochondrial dehydrogenase reduced the tetrazolium salt XTT to formazan salt, resulting in colorimetric change that correlated with cell viability according to the formula:

$$\mathrm{Inhibitory}\%\;=\;\left[1-\;\left(A\;/\;A^{\prime }\right)\right]\;\times \;100.$$

where A and A′ were referred to as the absorbance obtained from treated and control experiments at 492 nm, respectively using ELISA (Microplate Reader, BioTek Instruments Inc., Winooski, VT, USA) at 492 nm [72].

### 3.7. Anti-Cancers Activity of Z. coccineum

The aq.-ethanolic extract of *Z. coccineum* and its fractions were evaluated for their anti-cancer potentials against three cancer cell lines; MCF-7 breast cancer, HCT116 colon cancer, and HepG2 hepatoma cell lines (VACSERA- Cell Culture Unit, Cairo, Egypt) cultured in RPMI-1640 medium containing 10% FBS according to the standard procedure [73]. The cells were seeded in a 96-well plate one day before treatment with the extracts. Serial dilutions from tested extracts were added to well-plate cells after attachment of the cells to the well’s wall. Three independent assays were conducted for each dose. Treated cells with different extracts concentrations were incubated for 48 h (37 °C, 5% CO_2_), and staining with sulforhodamine B (SRB) for 10 min at 37 °C in dark. A solution of acetic acid (1%) was then used to wash the excess of the staining material. Tris base (10 nM, pH 7.4, 50 µL/well) was used to solubilize the dye, and plates were put under shaking for 5 min. Optical density was measured in an ELISA reader at 570 nm. The exponential curves for the cell viabilities against extracts concentrations, and Sigmaplot^®^ software (Systat^®^ Software Inc. Hounslow, UK) were used to calculate the IC_50_ values for each extract.

### 3.8. Topoisomerase-II β Inhibitory Effect of Z. coccineum

Topoisomerase-IIβ inhibitory effect of the aq.-ethanolic extract was measured using a human DNA Topoisomerase-IIβ (*h*-TOP-IIβ) ELISA Kit (Catalog # MBS942146, BioSource Inc., San Diego, CA, USA) according to the reported method [74]. The manufacturer’s instruction for working standards and sample preparation procedures was followed in full. To each well, 100 µL of the aq.-ethanolic extract, and the standard working solution were added and incubated at 37 °C for 2 hr. Then, the liquid was removed without washing. Biotin-conjugated antibody specific for *h*-TOP-IIβ was added to each well (100 µL) followed by a 1 h incubation at 37 °C. The contents of each well were washed three times and incubated for 1 h at 37 °C with avidin conjugated horseradish peroxidase (HRP) (100 µL). The wells were then aspirated and washed 5 times with washing buffer (200 µL) to remove any unbound avidin-enzyme substance. Accurately, 90 µL of 3,3′,5,5′-tetramethylbenzidine (TMB) liquid substrate was added to each well and the plate was light protected and incubated at 37 °C for 30 min before addition of stop solution (50 µL), and was thoroughly mixed. Optical density was measured at 450 nm within 5 min after stop solution addition.

### 3.9. Docking Studies

The X-ray crystallographic structures of Staph GyraseB 24 kDa in complex with Novobiocin (PDB ID: 4URO) and human topoisomerase-IIβ (*h*-TOP-IIβ) in complex with DNA and etoposide (PDB ID: 3QX3) were obtained from the Protein Data Bank, validated (rmsd = 0.21 A^0^) and prepared for docking. The docking simulations were performed using Molecular Operating Environment (MOE) version 2019.0101, Chemical Computing Group Inc., Montreal, QC, Canada. The subjected compounds (major identified compounds in *Z. coccineum*) were constructed in a 2D model using ChemDraw software then copied into the MOE interface where the energies of the proposed compounds were minimized to obtain the most stable conformers which were saved into a database; the latter was used in docking. The obtained ligand–enzyme complex model was then used in calculating the energy parameters using MMFF94x force field energy calculation and predicting the ligand–enzyme interactions at the active site.

## 4. Conclusions

The chemical constituents profiling through LC-MS analysis of *Z. coccineum* was achieved, and thirty-eight compounds belonging to different chemical classes from the mother liquor, aq.-ethanolic extract were identified. The presence of flavonoid glycosides was considered to exhibit synergistic anti-cancer and anti-microbial biofilm eradication activities. The in silico energy estimations, molecular docking simulations, and the energy requirements at the active site of the receptors, i.e., Staph GyraseB, and *h*-TOP-IIβ, for the plant’s major constituents’ identified in this study, together with the experimental observations of the anti-cancer and anti-microbial activity of the extract, suggested for further study to isolate, purify, and activity test the responsible active constituent(s) in near future. The discovery and confirmation of the plant’s constituent(s) involved in the biofilm obliteration to provide the anti-microbial activity agent(s) through the process of separation-purification and the chemical structure characterization, has the potential to yield new chemical leads and molecular templates for further development against the solo and multi-drug resistance microbes. The plan’s extension to bioassay-guided purification of the components from the extract leading to the identification of active constituent(s) as potent anti-cancer molecular lead has the prospective to provide new drug candidate, and newer molecular template which with the probabilities for structural modifications holds promise for further drive to culminate in natural products based new anti-cancer agent.

## Figures and Tables

**Table 1:** Secondary metabolites identified in the aq.-ethanolic extract of *Z. coccineum* by LC–ESI-TOF–MS.

No.	*RT* (min)	% *	Adduct Ion	Molecular Formula, Precursor *m*/*z*	Error (ppm)	Characteristic Fragments	Compounds Identity **	Refs.
1.	1.13	0.945	[M + H]^+^	C_10_H_26_N_4_ 203.2236	−1.0	112 [M + H − (CH_2_)_3_N_2_H_4_ − NH_3_]^+^,129 [M + H − (CH_2_)_3_N_2_H_4_]^+^, 203 [M + H]^+^	Spermine	[39,40]
2.	1.14	0.142	[M + H]^+^	C_7_H_19_N_3_ 146.1667	−8.3	129 [M + H − NH_2_]^+^, 146 [M + H]^+^	Spermidine	[40]
3.	1.46	0.026	[M − H]^−^	C_8_H_8_O_3_ 151.0601	0.6	107 [M − H − CO_2_]^−^, 151 [M − H]^−^	2-Hydroxyphenyl acetic acid	[46,47]
4.	1.47	0.458	[M − H]^−^	C_9_H_8_O_4_ 179.0572	1.3	135 [M − H − CO_2_]^−^, 150 [M − H − CO]^−^, 161 [M − H − H_2_O]^−^, 179 [M − H]^−^	Caffeic acid	[48]
5.	1.52	0.072	[M − H]^−^	C_7_H_6_O_3_ 137.0228	6.0	93 [M − H − CO_2_]^−^, 137 [M − H]^−^	Salicylic acid	[49]
6.	1.84	0.284	[M − H]^−^	C_9_H_8_O_3_ 163.0384	6.9	119 [M − H − CO_2_]^−^, 163 [M − H]^−^	*p*-Coumaric acid	[50]
7.	1.84	0.242	[M − H]^−^	C_21_H_24_O_9_ 419.0877	−0.7	257 [M – H-glucose]^−^, 419 [M − H]^−^	Isorhapontin	[42]
8.	2.48	0.069	[M − H]^−^	C_10_H_7_NO_3_ 188.0342	3.7	144 [M − H − CO_2_]^−^, 188 [M − H]^−^	Kynurenic acid	[41]
9.	3.28	0.389	[M − H]^−^	C_7_H_6_O_3_ 137.0228	5.3	93 [M − H − CO_2_]^−^, 137 [M − H]^−^	*p*-Hydroxybenzoic acid	[51]
10.	5.08	0.162	[M − H]^−^	C_15_H_20_O_4_ 263.131	−0.4	153 [M − H − C_6_H_6_O_2_]^−^, 219 [M − H − COO]^−^, 263 [M − H]^−^	Abscisic acid	[43]
11.	5.11	0.233	[M − H]^−^	C_15_H_12_O_7_ 303.0924	0.0	285 [M − H − H_2_O]^−^, 303 [M − H]^−^	Taxifolin	[52]
12.	5.86	0.135	[M − H]^−^	C_21_H_18_O_11_ 445.0773	2.8	269 [M − H − glucuronic acid]^−^,445 [M − H]^−^	Baicalin (Baicalein-7-*O*-glucuronide)	[53]
13.	6.08	0.082	[M − H]^−^	C_21_H_20_O_12_ 463.0886	−0.4	301 [M – H-glucose]^−^, 463 [M − H]^−^	Spiraeoside (Quercetin-4′-*O*-glucopyranoside)	[54]
14.	6.13	0.583	[M − H]^−^	C_19_H_24_O_5_ 331.1323	4.3	286 [M − H − OH]^−^, 331 [M − H]^−^	Gibberellin-A4	[44]
15.	6.21	0.101	[M]^+^	C_27_H_31_O_15_ 595.163	3.6	287 [M − rutinose]^+^, 449[M − rhamnose]^+^, 595 [M]^+^	Cyanidin-3-*O*-rutinoside	[55]
16.	6.29	0.084	[M + H]^+^	C_9_H_10_O_4_ 183.0916	−0.4	123 [M + H − CH_3_ − CO_2_H]^+^, 140 [M + H − CH_3_ − CO]^+^, 168 [M + H − CH_3_]^+^, 183 [M + H]^+^	Syringaldehyde	[40]
17.	6.32	0.076	[M + H]^+^	C_12_H_10_O_4_ 219.1468	6.7	161 [M + H − CH_3_ − CH_3_CO]^+^, 219 [M + H]^+^	7-Acetoxy-4-methyl coumarin	[45]
18.	6.47	0.019	[M + H]^+^	C_15_H_12_O_5_ 273.1365	0.6	147 [M + H − C_6_H_6_O_3_]^+^, 273 [M + H]^+^	Naringenin	[56]
19.	6.51	0.661	[M + H]^+^	C_27_H_30_O_16_ 611.1602	0.8	153, 229 [M + H − rutinose − H_2_O − 2CO]^+^, 303 [M + H − rutinose]^+^ 465 [M + H − rhamnose]^+,^ 611 [M + H]^+^	Rutin	[28]
20.	6.52	0.153	[M + H]^+^	C_15_H_10_O_7_ 303.049	3.0	153,165,229 [M + H − H_2_O − 2CO]^+^, 247 [M + H-2CO]^+^, 257 [M + H − H_2_O − CO]^+^, 285 [M + H − H_2_O]^+^, 303 M + H]^+^	Quercetin	[28]
21.	6.53	19.807	[M − H]^−^	C_30_H_26_O_13_ 593.1541	−1.1	285 [M − H, 3-O-(6′′′′-*p*-coumaroyl)-glucoside]^−^, 593 [M − H]^−^	Tiliroside	[57]
22.	6.55	0.070	[M − H]^−^	C_11_H_12_O_5_ 223.1339	0.5	179 [M − H − CO_2_]^−^, 223 [M − H]^−^	Sinapic acid	[58]
23.	6.79	0.773	[M − H]^−^	C_42_H_66_O_15_ 809.4398	9.2	603 [M − H-glucose-CO_2_]^,^ 647 [M − H-glucose]^−^, 809 [M − H]^−^	Zygophyloside-K	
24.	6.84	1.618	[M − H]^+^	C_27_H_30_O_14_ 579.144	2.7	287 [M + H-2-rhamnose]^+^, 433 [M + H-rhamnose]^+^, 579 [M + H]^+^	Kaempferol 3,7-di-O-α-L-rhamnoside	[59]
25.	7.08	0.077	[M − H]^−^	C_28_H_32_O_16_ 623.2877	−5.0	300 [M − H-rutinose-CH_3_]^−^, 315 [M − H-rutinose]^−^, 623 [M − H]^−^	Isorhamnetin-3-O-rutinoside	[60]
26.	7.11	0.084	[M + H]^+^	C_21_H_20_O_12_ 465.1458	4.8	229 [M + H-glactose-H_2_O-2CO]^+^, 247 [M + H-glactose-2CO]^+^, 303 [M + H-galactose]^+^, 465 [M + H]^+^	Hyperoside(Quercetin-3-O-galactoside)	[28,61]
27.	7.11	1.489	[M + H]^+^	C_15_H_10_O_6_ 287.0546	0.8	137, 153, 241 [M + H − H_2_O − CO]^+^, 269 [M + H − H_2_O]^+^, 287 [M + H]^+^	Luteolin	[28]
28.	7.26	4.752	[M + H]^+^	C_22_H_22_O_12_ 479.1185	1.0	302 [M + H-glucose-CH_3_]^+^, 317 [M + H-glucose]^+^, 479 [M + H]^+^	Isorhamnetin-3-O-glucoside	[62]
29.	7.49	9.674	[M − H]^−^	C_42_H_66_O_18_S 889.3969	0.0	97 [SO_4_H]^−^, 683 [M − H-Glc-CO_2_]^−^, 727 [M − H-Glc]^−^, 845 [M − H-CO_2_]^−^, 889 [M − H]^−^	Zygophyloside-G	[36]
30.	7.86	0.394	[M + H]^+^	C_9_H_8_O 133.1006	0.3	105 [M + H-CO]^+^, 115 [M + H − H_2_O]^+^, 133 [M + H]^+^	Cinnamaldehyde	[40]
31.	8.03	12.786	[M − H]^−^	C_42_H_66_O_17_S 873.401	1.1	587 [M – H-glucose-CO_2_-SO_3_]^−^, 667 [M − H-glucose-CO_2_]^−^, 711 [M − H-glucose]^−^,873 [M−]^−^	Zygophyloside-F	[5]
32.	9.28	3.315	[M − H]^−^	C_42_H_66_O_14_ 793.4397	2.8	254 [M − H-glucose-(CO_2_)_2_]^−^, 587 [M − H-glucose-CO]^−^, 749, 793 [M − H]^−^	3-*O*-[β-D-Quinovo pyranosyl] quinovic acid-28-β-D-gluco pyranosyl ester	[6]
33.	9.41	0.367	[M + H]^+^	C_21_H_20_O_10_ 433.1104	4.3	165, 241 [M + H-H_2_O-CO]^+^, 287 [M + H-rhamnose]^+^, 433 [M + H]^+^	Kaempferol-3-O-α-L-rhamnoside	[28,61]
34.	10.13	0.559	[M − H]^−^	C_36_H_56_O_10_ 647.3752	−6.6	602 [M − H-CO_2_]^−^, 647 [M − H]^−^	3-*O*-[β-D-gluco pyranosyl] quinovic acid	[6]
35.	10.81	0.332	[M + H]^+^	C_15_H_10_O_5_ 271.0609	−0.5	119, 121, 153, 225 [M + H-H_2_O-CO]^+^, 271 [M + H]^+^	Apigenin	[28]
36.	11.14	0.111	[M + H]^+^	C_16_H_12_O_6_ 301.0688	4.8	153, 286 [M + H-CH_3_]^+^, 301 [M + H]^+^	Kaempferide	[63]
37.	11.30	0.294	[M + H]^+^	C_16_H_12_O_7_ 317.0638	5.0	121, 153, 229, 302 [M + H-CH_3_]^+^, 317 [M + H]^+^	Isorhamnetin	[28]
38.	11.87	0.034	[M − H]^−^	C_16_H_12_O_4_ 267.0668	0.4	204, 251, 252 [M − H-CH_3_]^−^, 267 [M − H]^−^	Formononetin	[64]

* Relative percentages of the identified compounds in the plant extracts were calculated based on the total peak area in the chromatogram, ** Tentative identity.

**Table 2:** Anti-microbial Activity of *Z. coccineum* aq.-ethanolic extract and fractions.

Tested Samples	aq.-Ethanolic Extract			Chloroform Fraction			Ethyl Acetate Fraction			*n*-Butanol Fraction			Amphotericin B		
**Fungi**	MIC	MBIC	MBEC	MIC	MBIC	MBEC	MIC	MBIC	MBEC	MIC	MBIC	MBEC	MIC	MBIC	MBEC
*Aspergillus fumigatus*	15.63	1.95	125	1.95	0.49	15.63	3.9	0.49	62.5	31.25	3.9	NA	0.98	0.24	15.63
*Candida albicans*	62.5	3.9	250	0.98	0.24	7.81	3.9	0.98	31.25	125	31.25	1000	0.49	0.12	15.63
**Gram-Positive Bacteria**													**Ciprofloxacin**		
*Streptococcus pneumoniae*	125	31.25	NA	0.98	0.24	15.63	7.81	0.98	62.5	125	15.3	1000	0.98	0.12	31.25
*Staphylococcus aureus*	15.63	3.9	125	0.49	0.12	3.9	3.9	0.49	62.5	62.5	7.81	250	0.49	0.24	15.63
**Gram-Negative Bacteria**													**Ciprofloxacin**		
*Pseudomonas aeruginosa*	31.25	3.9	250	0.98	0.24	15.63	3.9	0.98	31.25	125	15.63	NA	1.95	0.24	31.25
*Escherichia coli*	15.63	3.9	125	1.95	0.24	15.63	0.98	0.49	15.63	31.25	3.9	250	0.98	0.12	31.25

All values are in µg/mL of the extracts, All microbial strains were of American type culture collection (ATCC), MIC: Minimum inhibitory concentration, MBIC: Minimum Biofilm Inhibitory Concentration, MBEC: Minimum Biofilm Eradication Concentration, NA: No activity.

**Table 3:** Topoisomerase-IIβ inhibition activities of *Z. coccienum* aq.-ethanolic extract and fractions *.

**Cell-Lines/Enzyme**	**aq.-Ethanolic Extract** **(IC_50,_ µg/mL)**	**Chloroform Fraction** **(IC_50,_ µg/mL)**	**Ethyl Acetate Fraction** **(IC_50,_ µg/mL)**	**n-Butanol Fraction** **(IC_50,_ µg/mL)**	**Staurosporine** **(IC_50_, ng/mL)**
MCF-7	3.47 ± 0.28	5.78± 0.56	8.43± 0.86	9.78 ± 0.98	
HCT-116	3.19 ± 0.29	4.12 ± 0.036	6.22 ± 0.056	10.17 ± 1.1	
HepG2	2.27 ± 0.26	3.17 ± 0.34	5.52 ± 0.52	8.26 ± 0.87	
Topoisomerase-IIβ (TOP-IIβ) inhibition activity	45.05 ng/mL				135.33 ng/mL

* Tests were conducted in triplicate and results are expressed as mean ± SD.

**Table 4:** *Z. coccineum* major constituents’ relative abundance in plant extract, and their in silico binding interaction energies.

Compound Name and Entry (#) in Table 1	Energy: ΔG (Kcal/mole) at 4URO	Energy: ΔG (Kcal/mole) at 3QX3
Tiliroside (**21**)	−6.46	−6.63
Zygophyloside-F (**31**)	−6.06	−6.22
Isorhamnetin-3-O-glucoside (**28**)	−6.38	−5.75
3-O-[β-D-quinovopyranosyl] quinovic acid- 28-β-D-glucopyranosyl ester (**32**)	−6.37	−6.23
Kaempferol 3,7-di-O-α-L-rhamnoside (**24**)	−6.47	−5.65
Luteolin (**27**)	−5.19	−5.08
Spermine (**1**)	−4.91	−5.10
Gibberellin-A4 (**14**)	−3.81	−4.23
Caffeic acid (**4**)	−4.60	−4.60
Etoposide (standard anti-cancer drug)		−5.94
Novobiocin (standard anti-microbial drug)	−6.72	

## Supplementary Materials

The followings are available online, Figures S1–S4: Chromatograms; Figures S5–S20: Mass spectra; Figures S21–S22: UV-calibration curves; Table S1: 3D structures, binding domains, and energies of the major constituents of *Z. coccineum*.
