# Supplementary material for: Phytochemical Profiling, In Vitro and In Silico Anti-Microbial and Anti-Cancer Activity Evaluations and Staph GyraseB and h-TOP-IIβ Receptor-Docking Studies of Major Constituents of Zygophyllum coccineum L. Aqueous-Ethanolic Extract and Its Subsequent Fractions: An Approach to Validate Traditional Phytomedicinal Knowledge
Source: Molecules. 2021 Jan 22;26(3):577. doi: 10.3390/molecules26030577 (PMC7866194; doi:10.3390/molecules26030577)
Supplement: Supplementary file 1 [file molecules-26-00577-s001.pdf]

__Supplementary Materials__

***Title*: Phytochemical profiling, *in vitro* and *in silico* anti-microbial and anti-cancer activity evaluations, and Staph-GyraseB and *h*-TOP-IIβ receptor-docking studies of major constituents of *Zygophyllum coccineum* L. aqueous-ethanolic extract and its subsequent fractions: An approach to validate traditional phytomedicinal knowledge**

A. CHROMATOGRAMS:

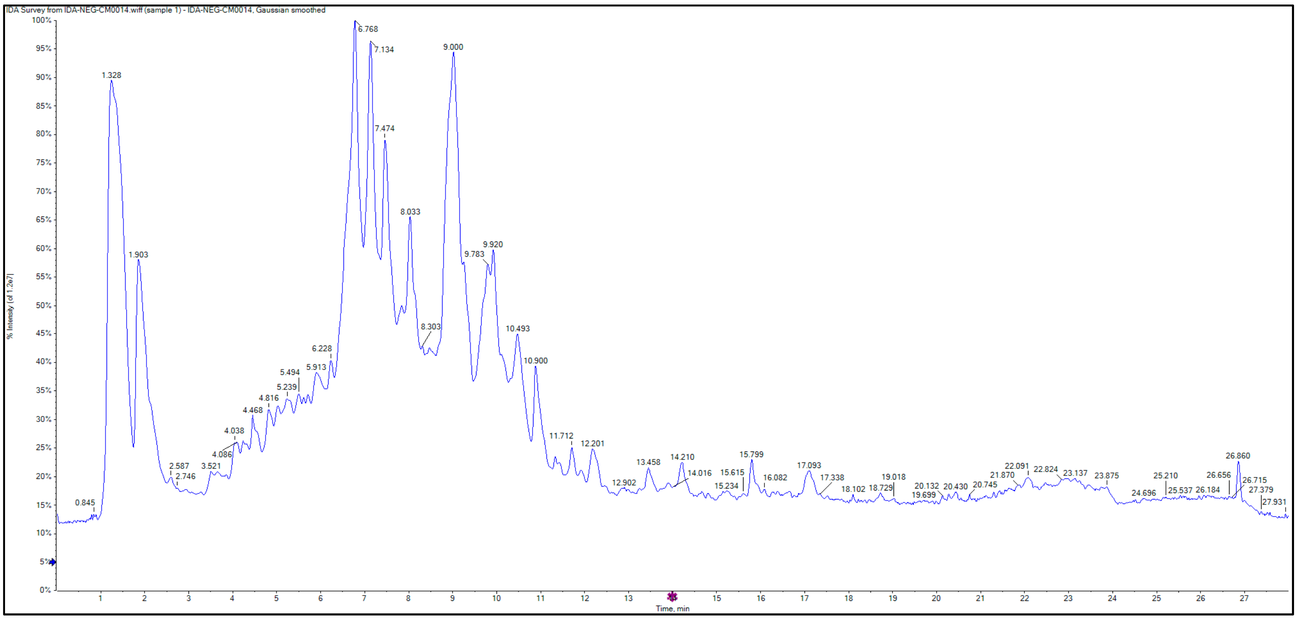

Figure S1: -ESI-TOF–MS negative ion mode mass analysis-LC Chromatogram of *Z. coccineum* mother liquor, aq.-ethanolic extract

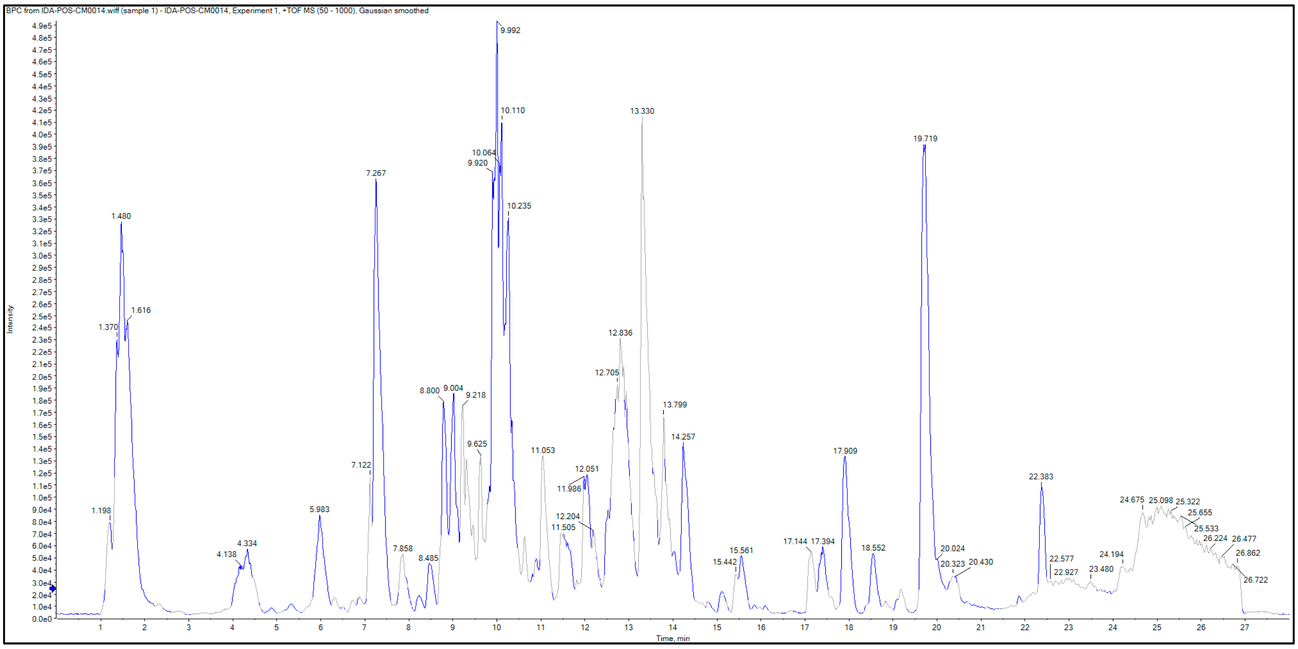

Figure S2: ESI-TOF–MS positive ion mode mass analysis-LC Chromatogram of *Z. coccineum* mother liquor, aq.-ethanolic extract

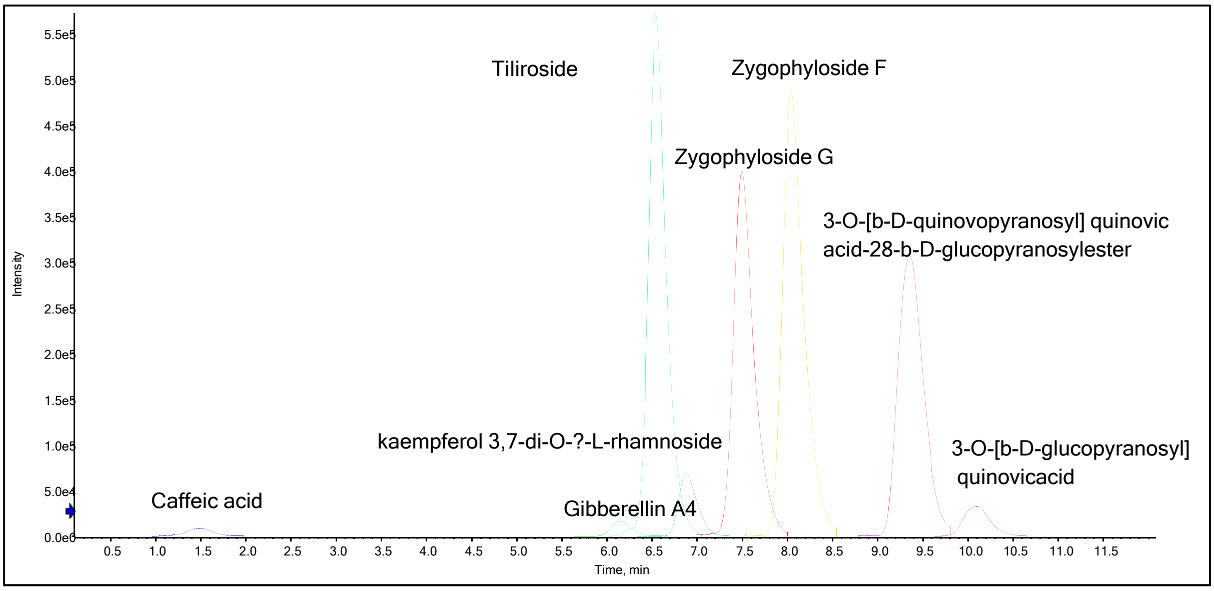

Figure S3: LC chromatogram of negative ion mode mass analysis for major constituents in *Z. coccineum* aq.-ethanolic extract

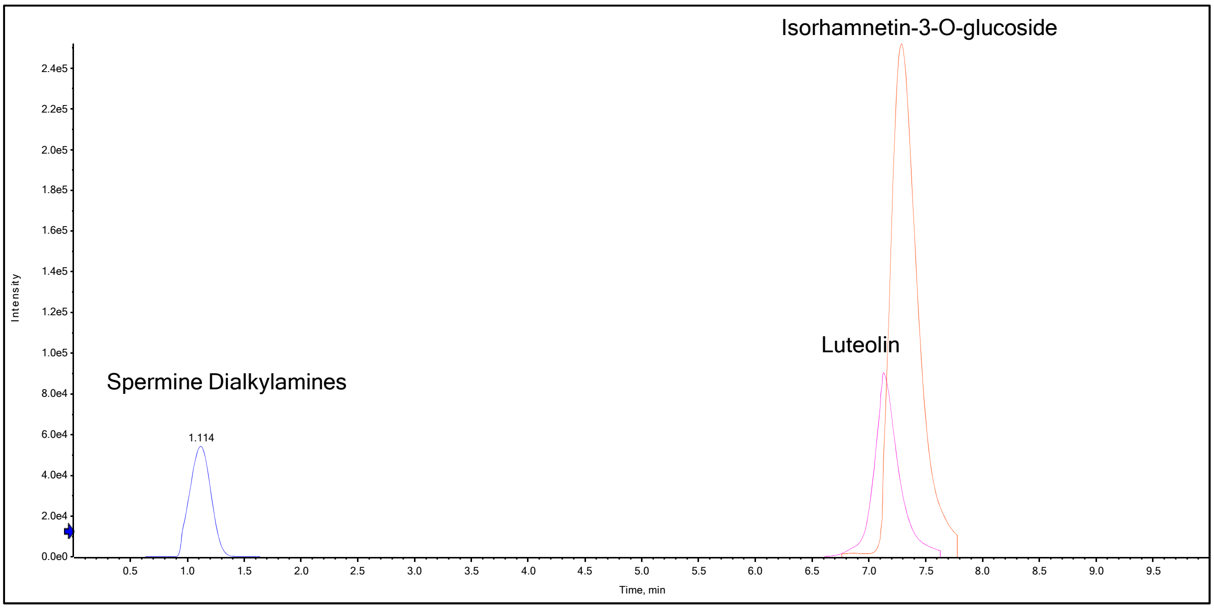

Figure S4: LC chromatogram of positive ion mode mass analysis for major constituents in *Z. coccineum* aq.-ethanolic extract

B. MASS SPECTRA:

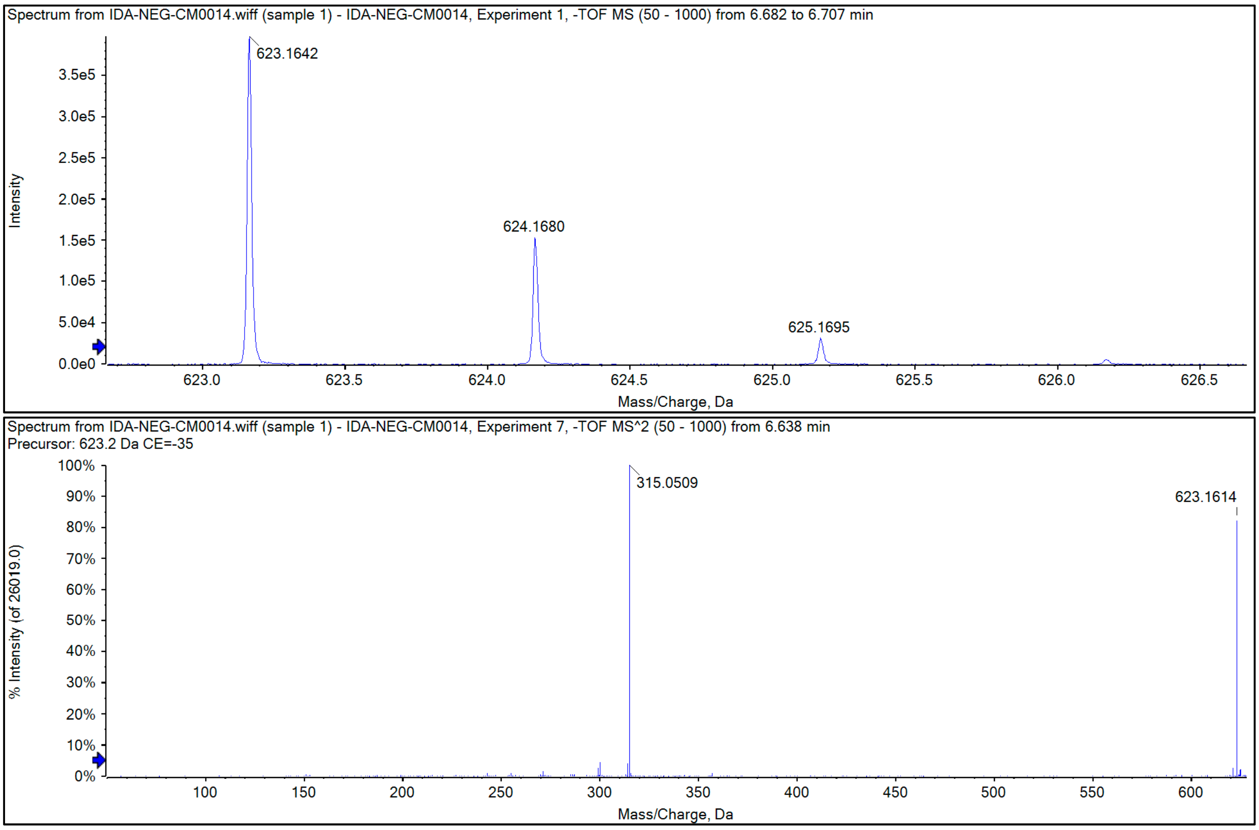

Figure 5S: Negative ion mode mass fragmentation of Isorhamnetin-3-O-rutinoside

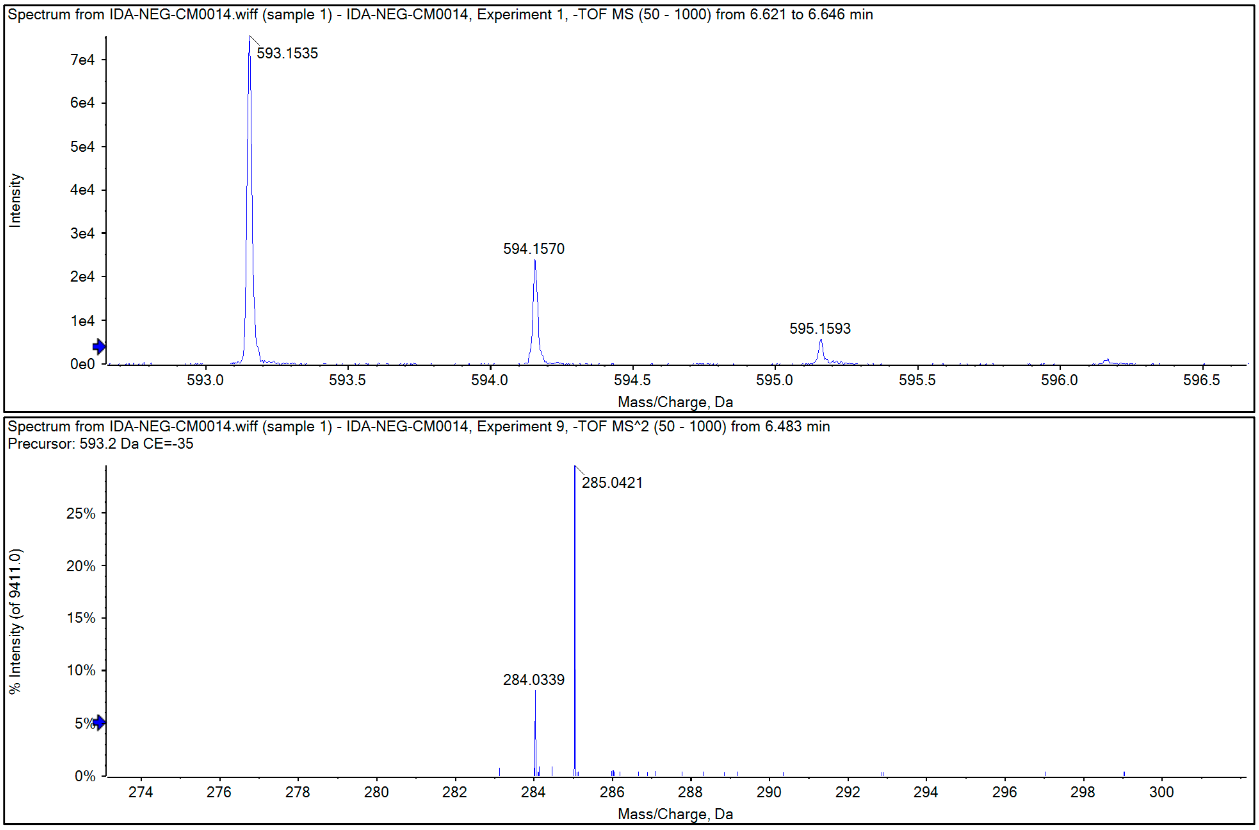

Figure 6S: Negative ion mode mass fragmentation of Kaempferol-3-O-(6''''-*p*-coumaroyl)-glucoside

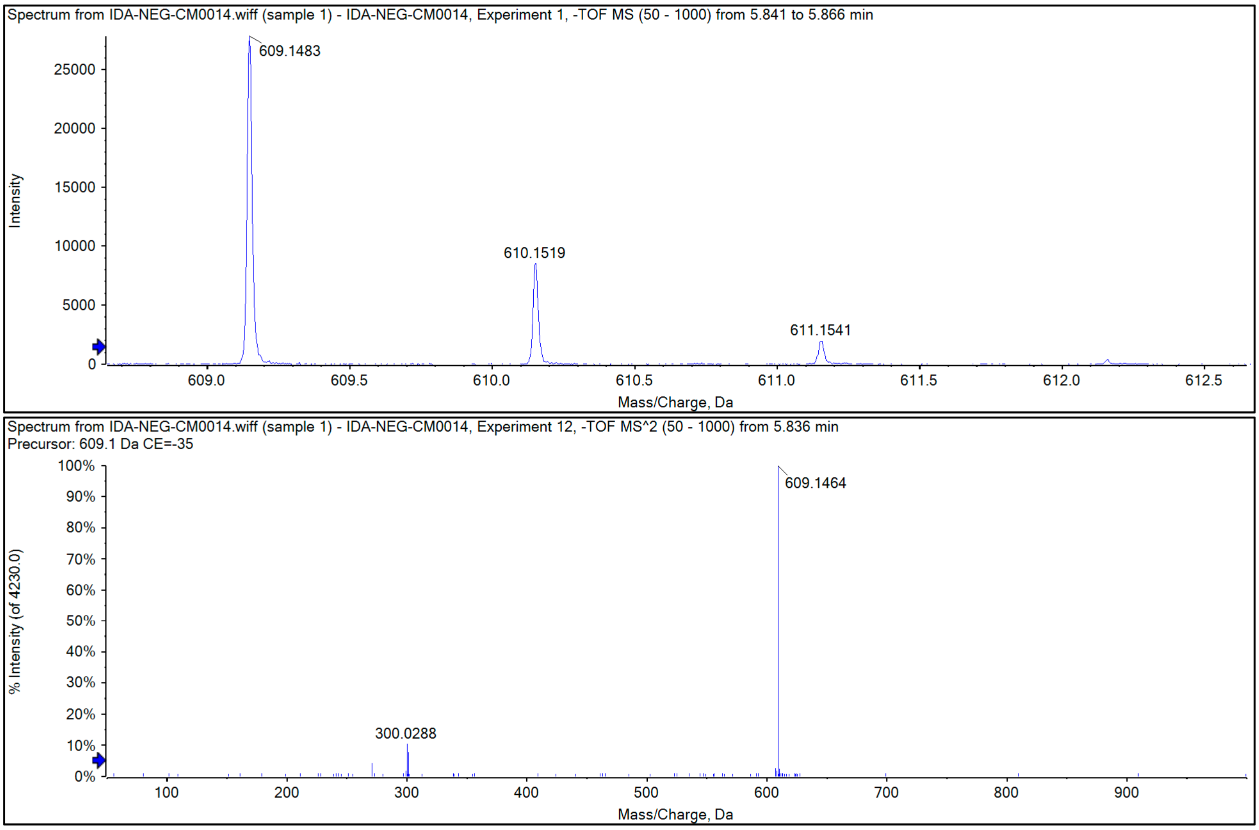

Figure 7S: Negative ion mode mass fragmentation of Delphinidin-3-O-(6''-O-α-rhamnopyranosyl-β-glucopyranoside)

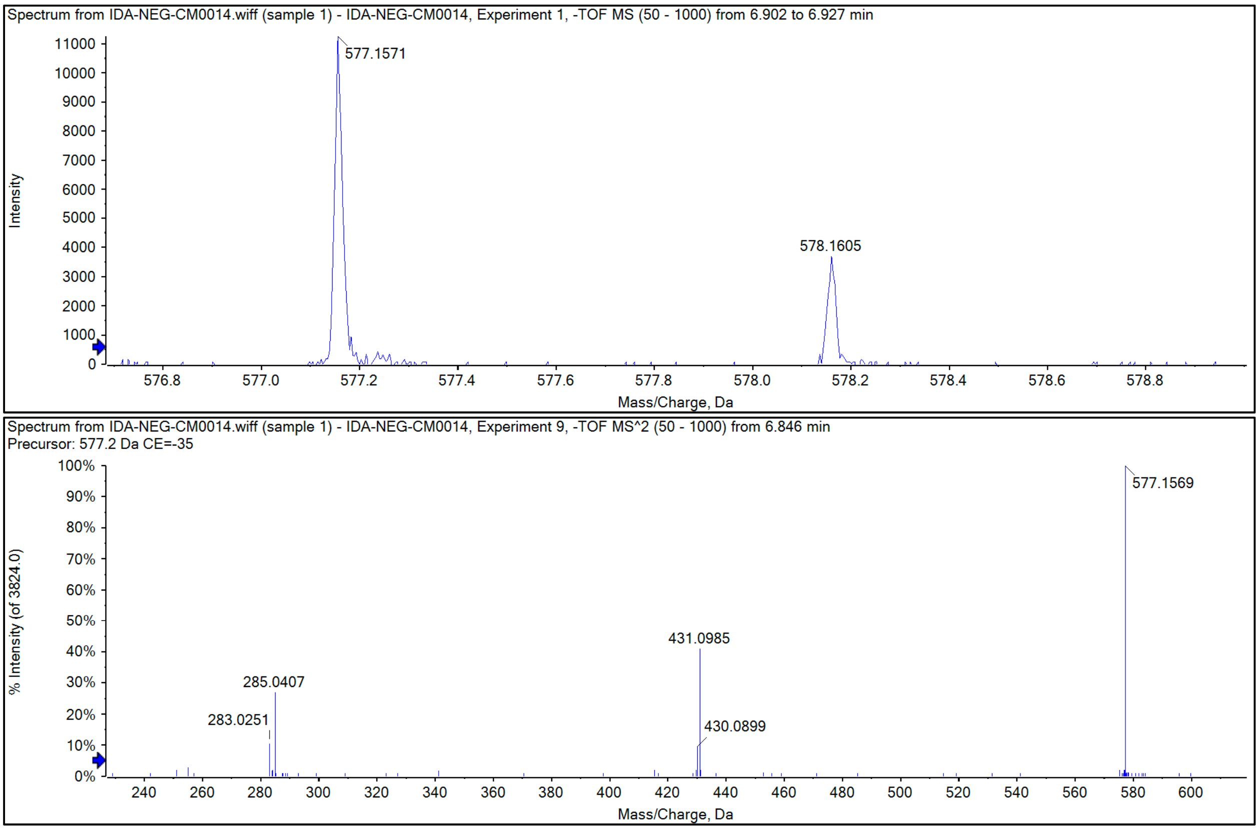

Figure 8S: Negative ion mode mass fragmentation of Kaempferol-3,7-O-bis-α-L-rhamnoside

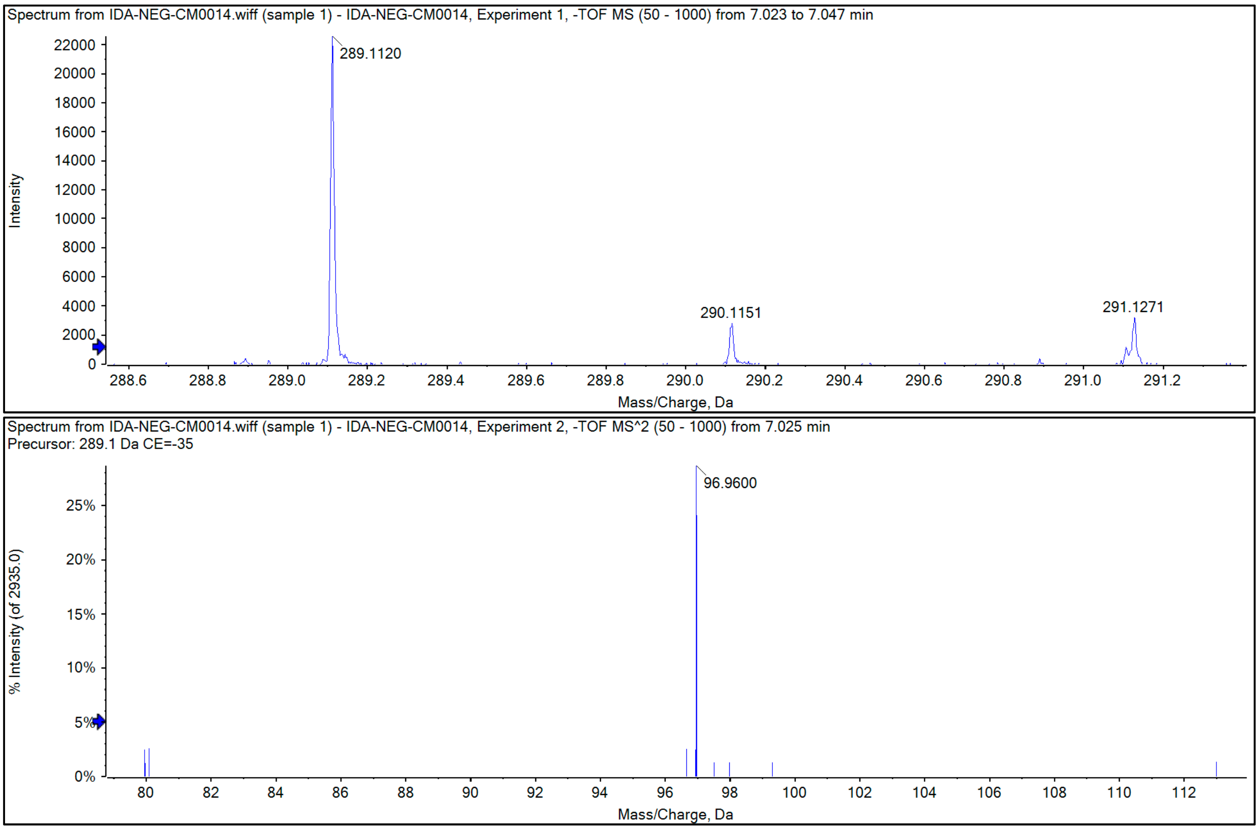

Figure 9S: Negative ion mode mass fragmentation of Quercetin

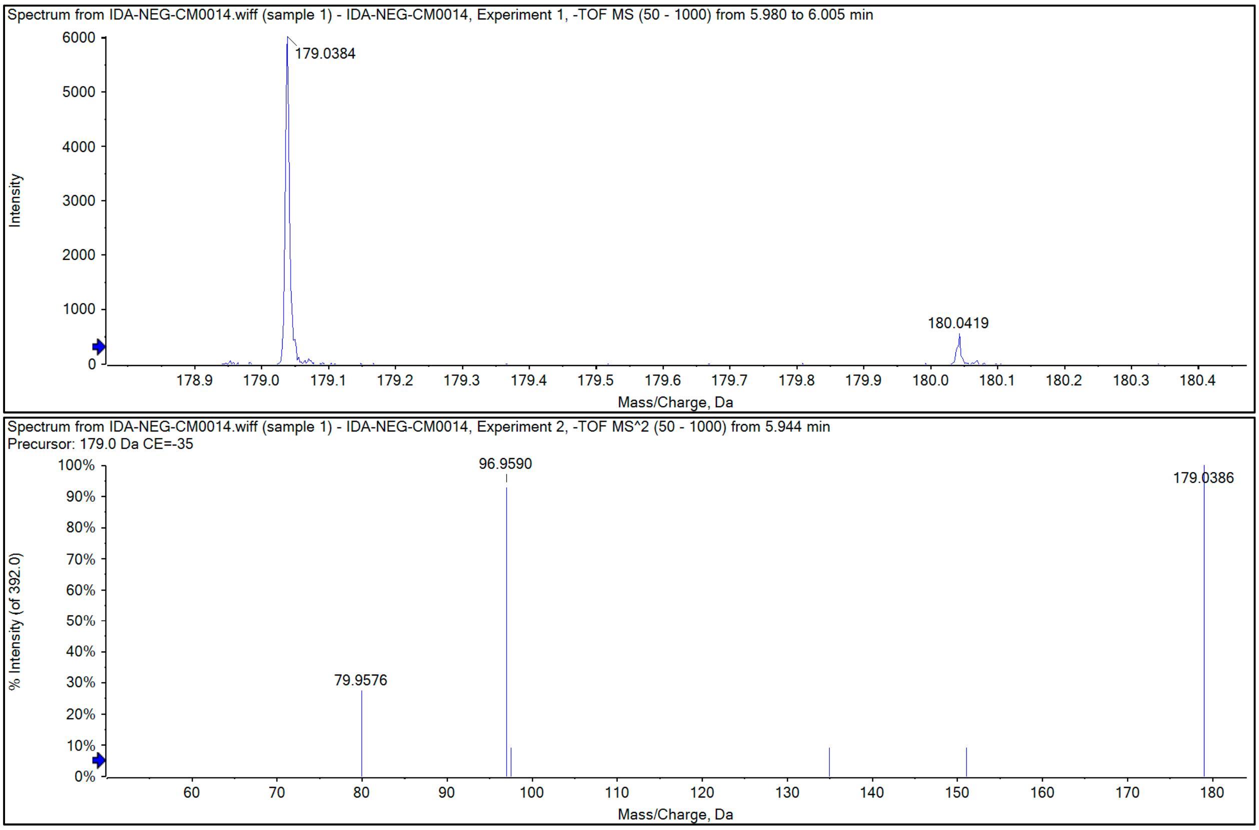

Figure 10S: Negative ion mode mass fragmentation of Caffeic acid

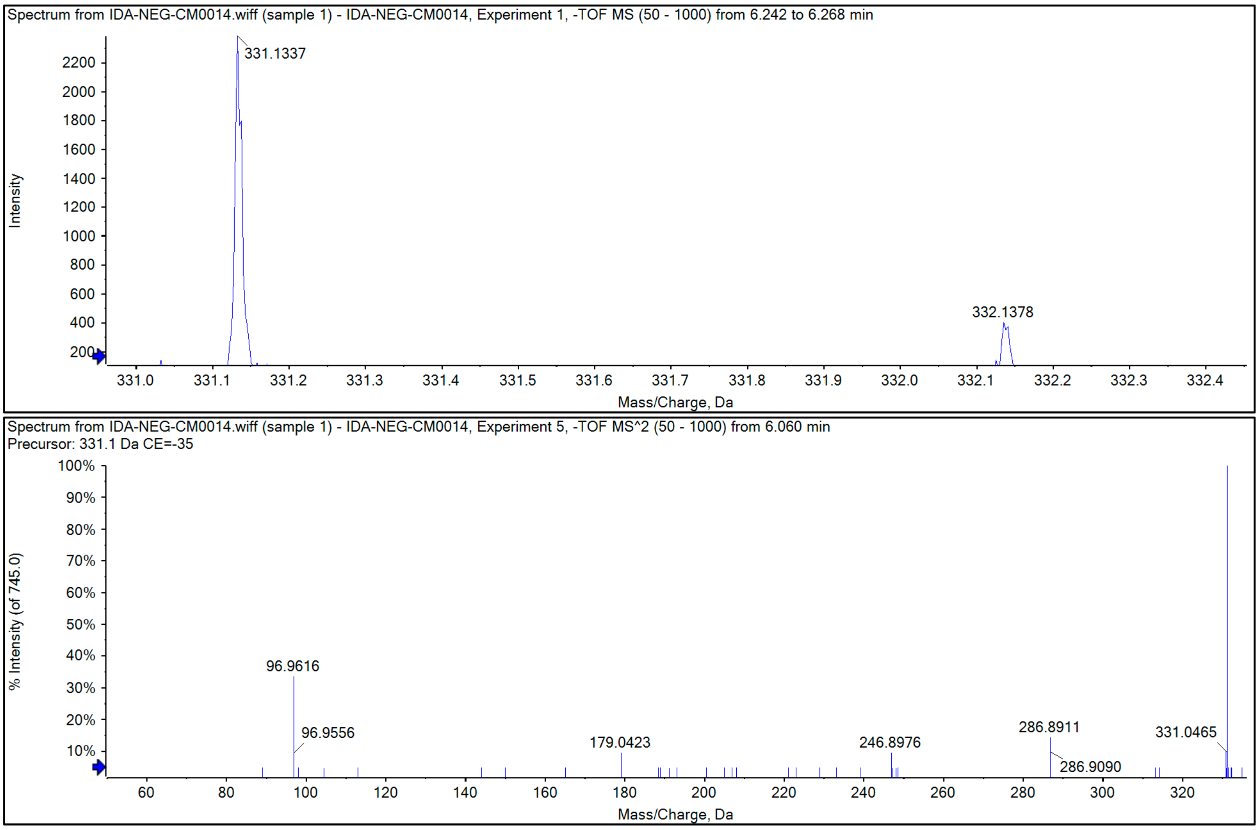

Figure 11S: Negative ion mode mass fragmentation of Gibberellin-A4

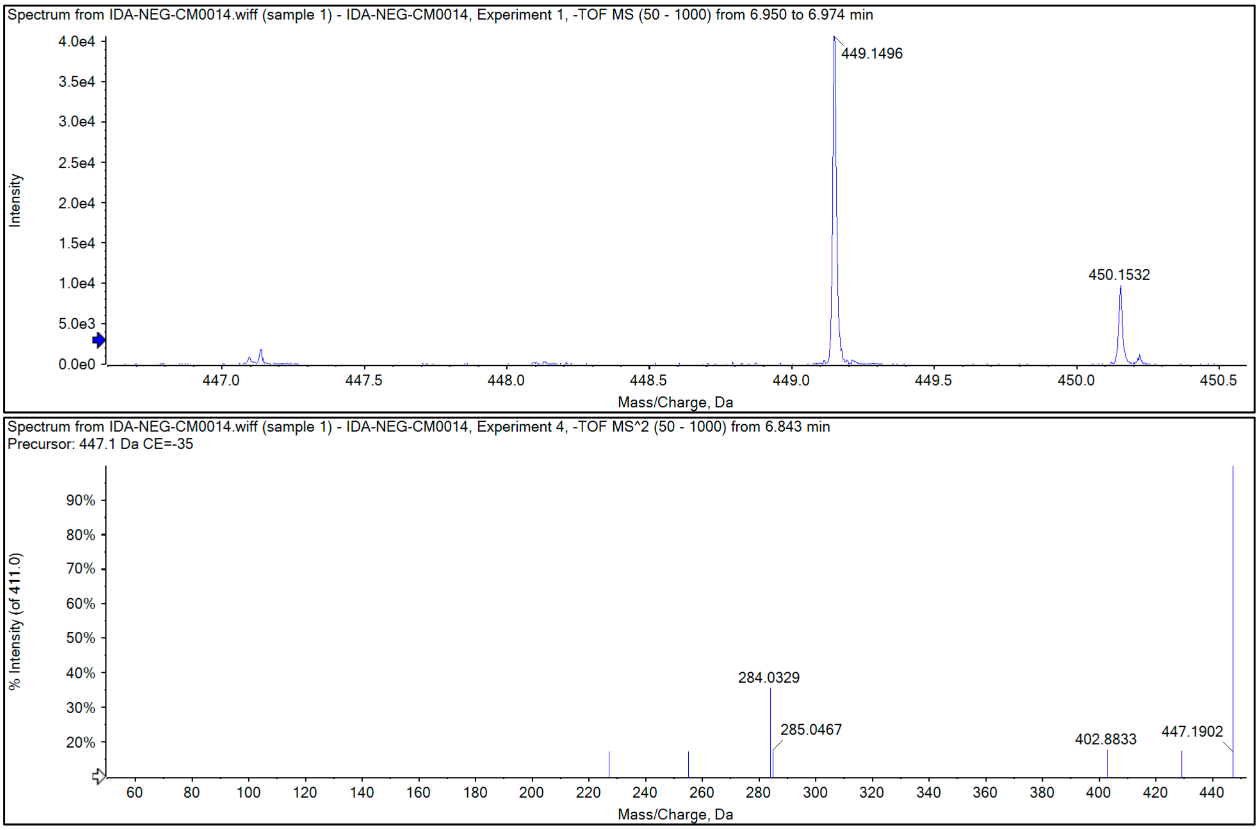

Figure 12S: Negative ion mode mass fragmentation of Kaempferol-3-O-glucoside

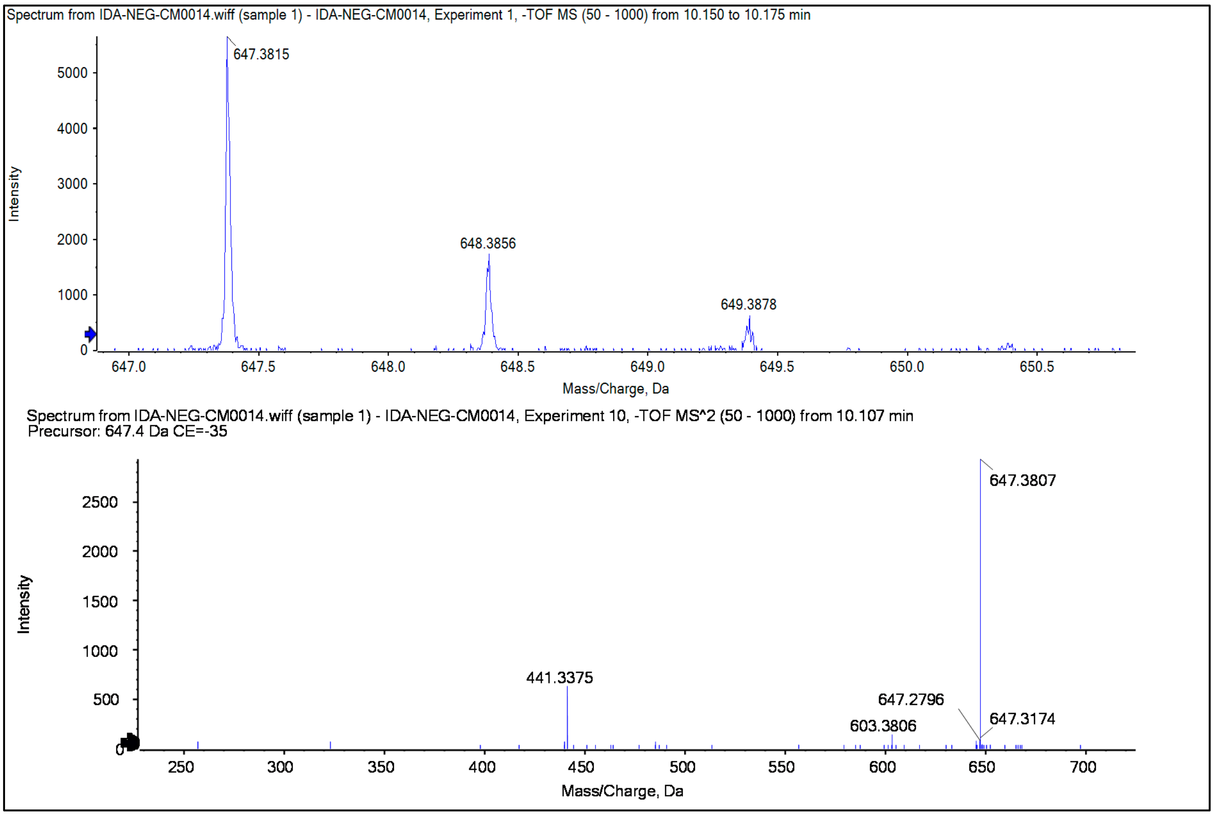

Figure 13S: Negative ion mode mass fragmentation of 3-O-[β-D-glucopyranosyl] quinovic acid

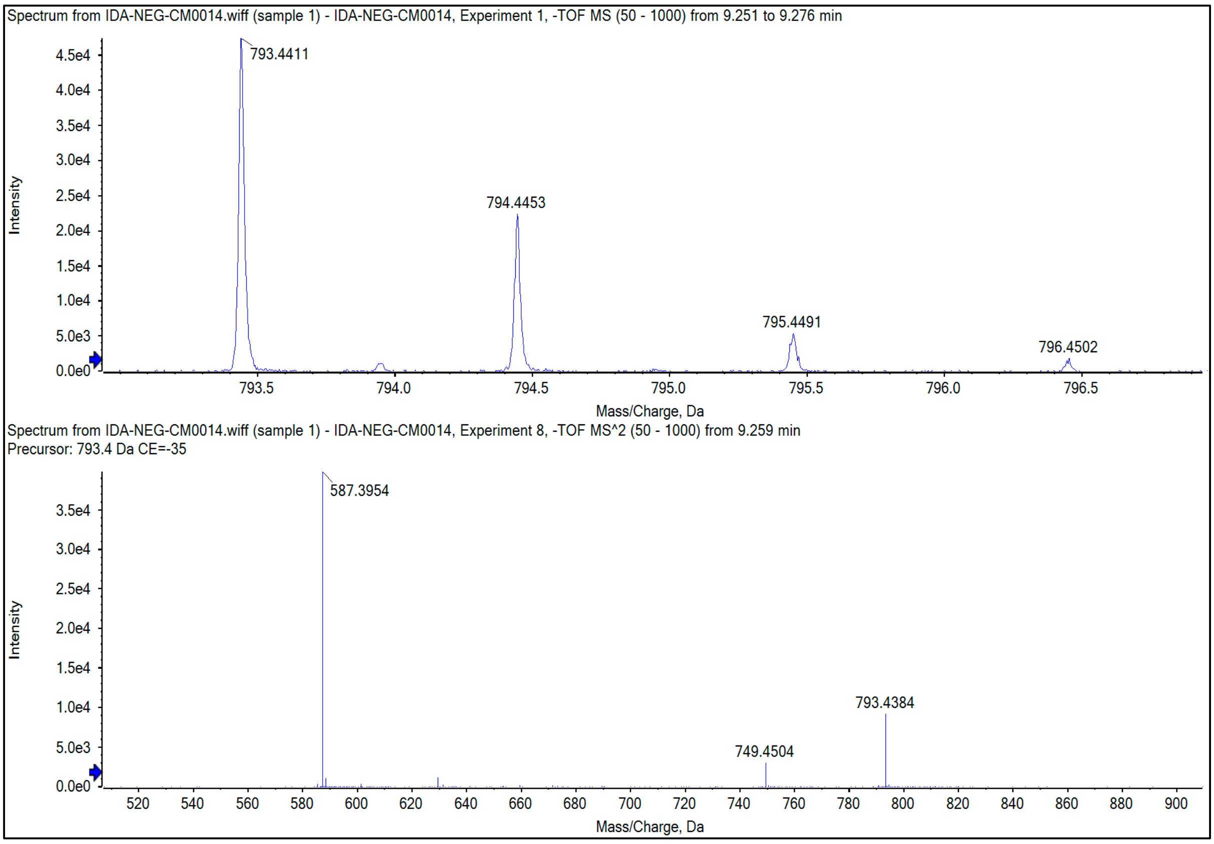

Figure 14S: Negative ion mode mass fragmentation of 3-O-[β-D-quinovopyranosyl] quinovic acid-28-β-D-glucopyranosyl ester

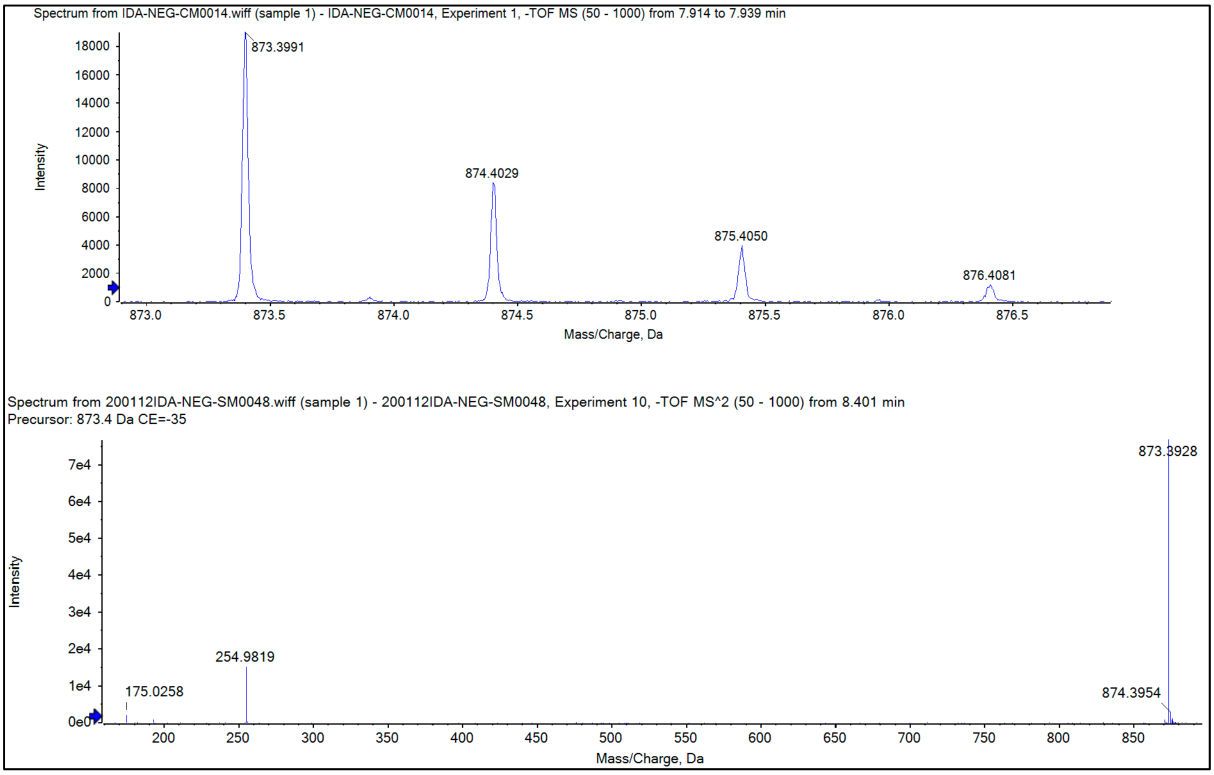

Figure 15S: Negative ion mode mass fragmentation of Zygophyloside-F

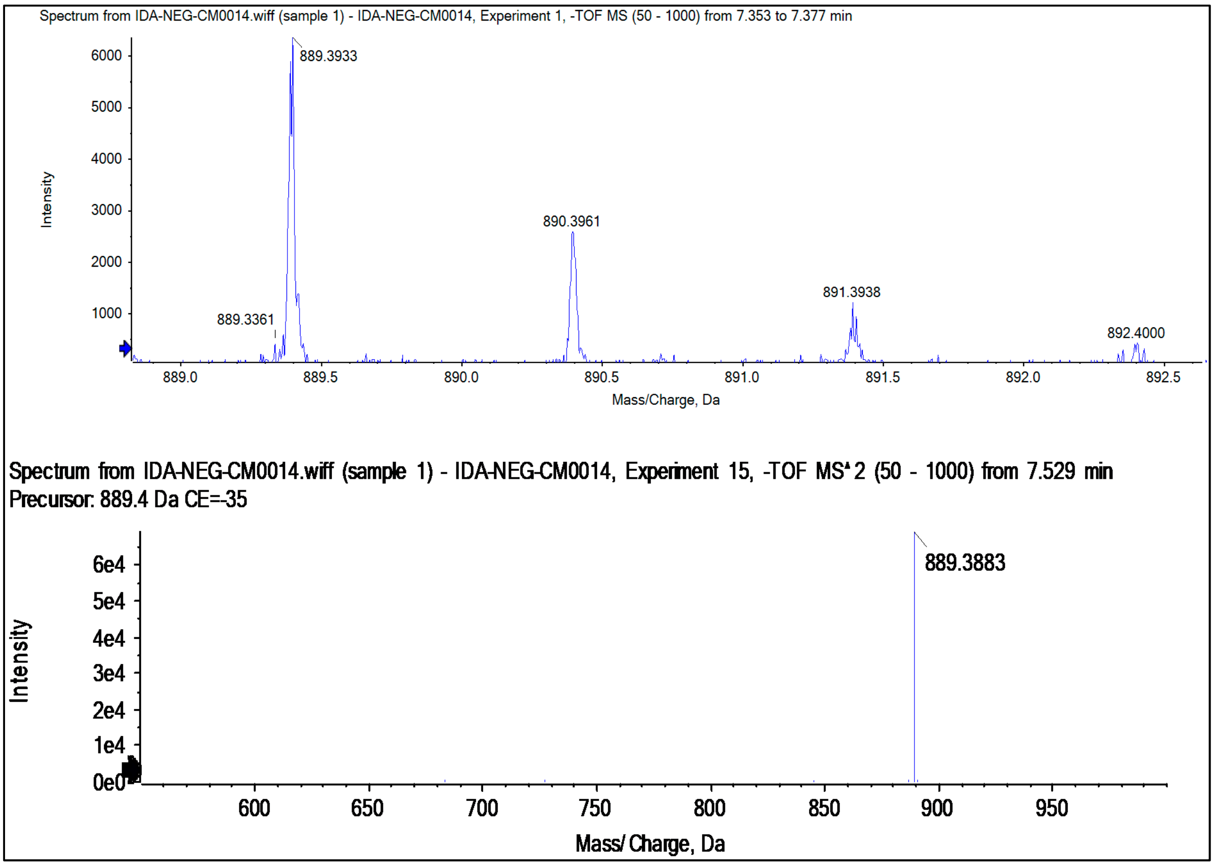

Figure 16S: Negative ion mode mass fragmentation of Zygophyloside-G

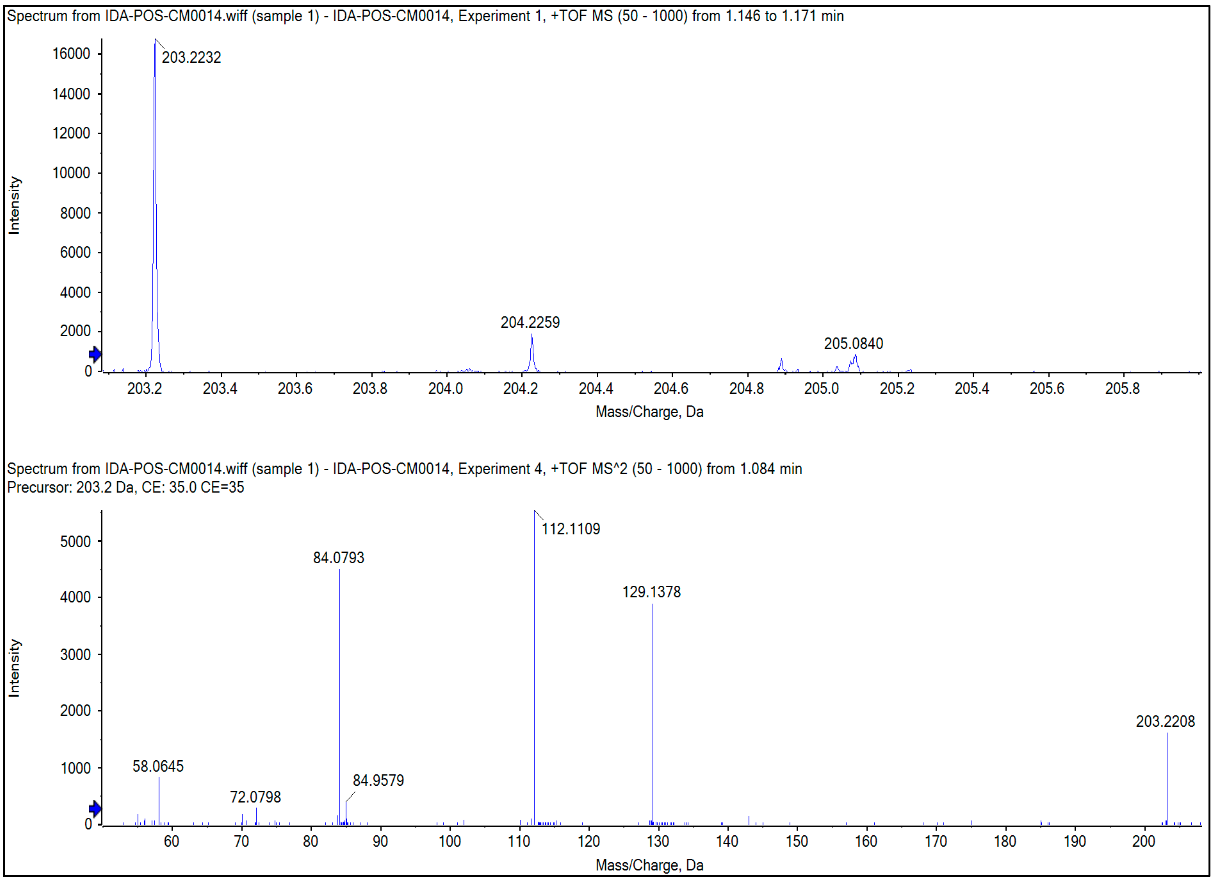

Figure 17S: Positive ion mode mass fragmentation of Spermine

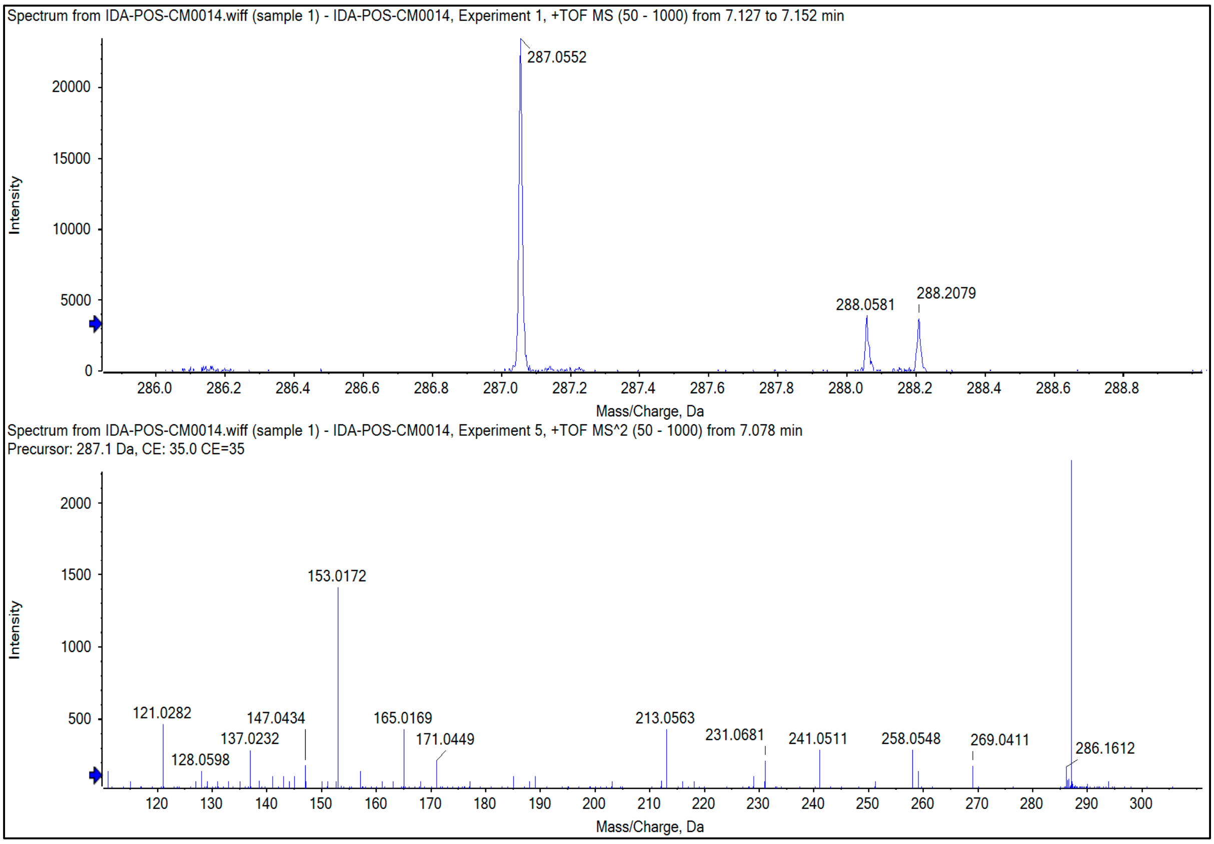

Figure 18S: Positive ion mode mass fragmentation of Luteolin

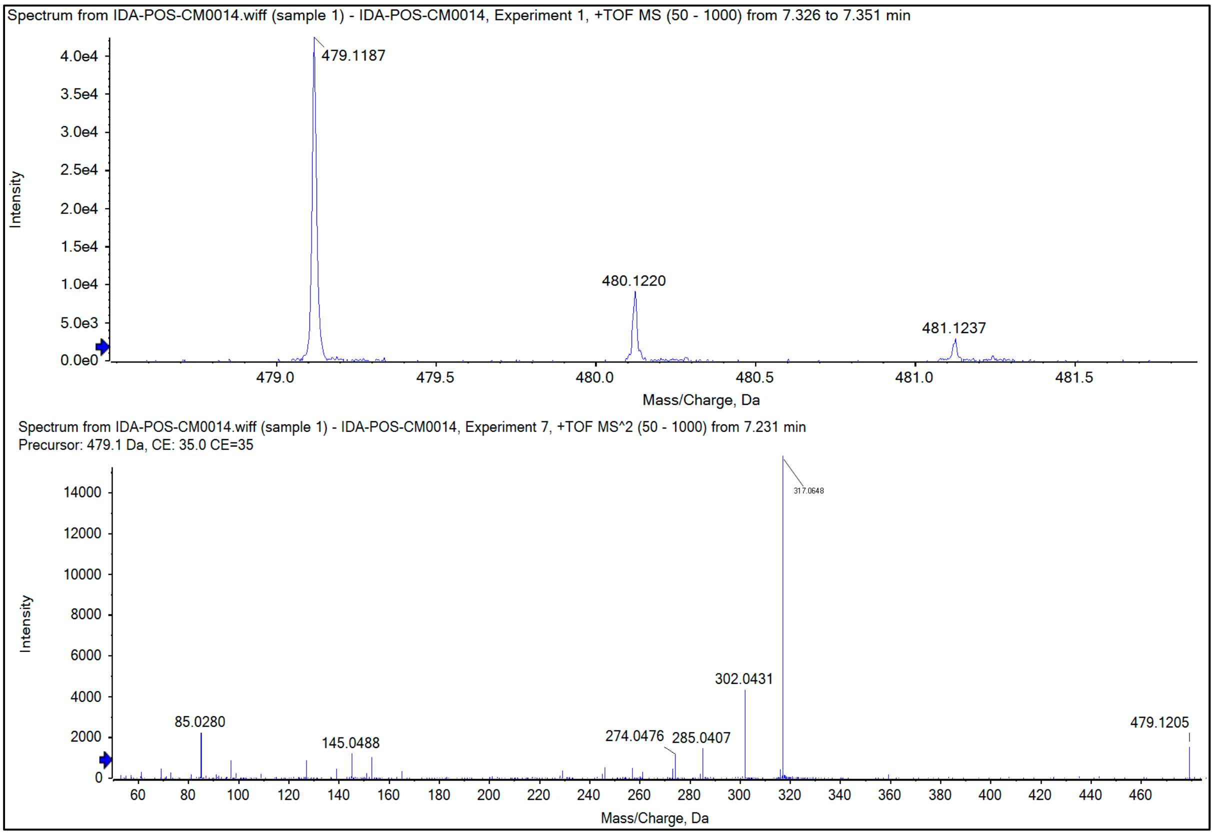

Figure 19S: Positive ion mode mass fragmentation of Isorhamnetin-3-O-glucoside

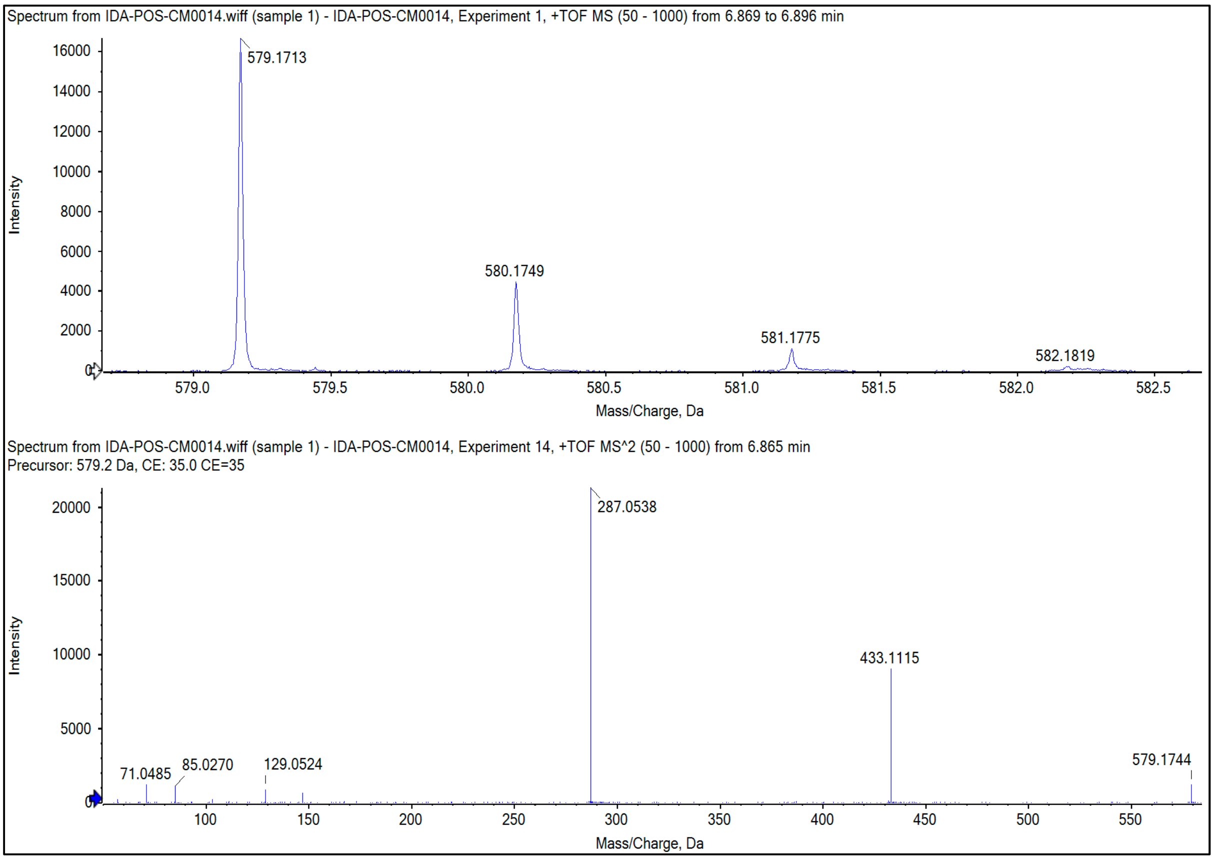

Figure 20S: Positive ion mode mass fragmentation of Kaempferol 3,7-di-O-α-L-rhamnoside

C. UV-CALIBRATION CURVES:

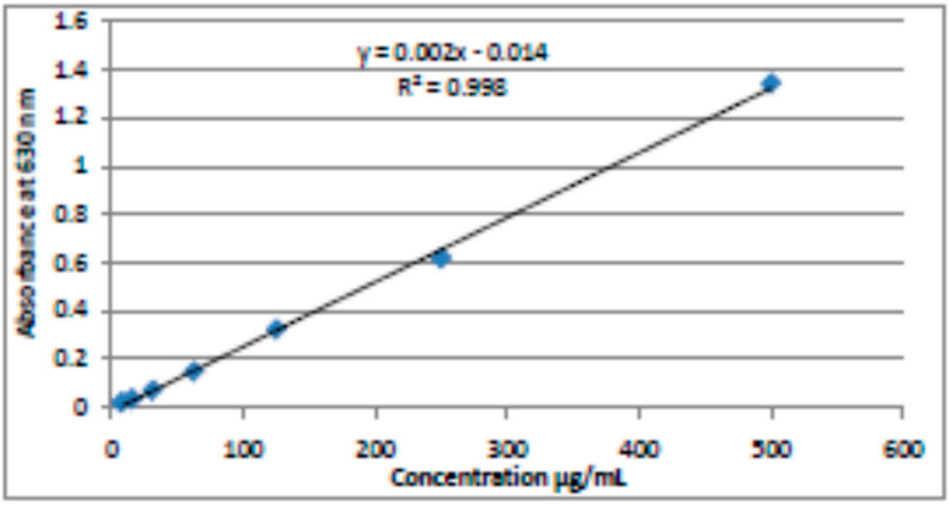

Figure 21S: Standard calibration curve of gallic acid used in the calculation of total phenolic contents of *Z. coccineum*

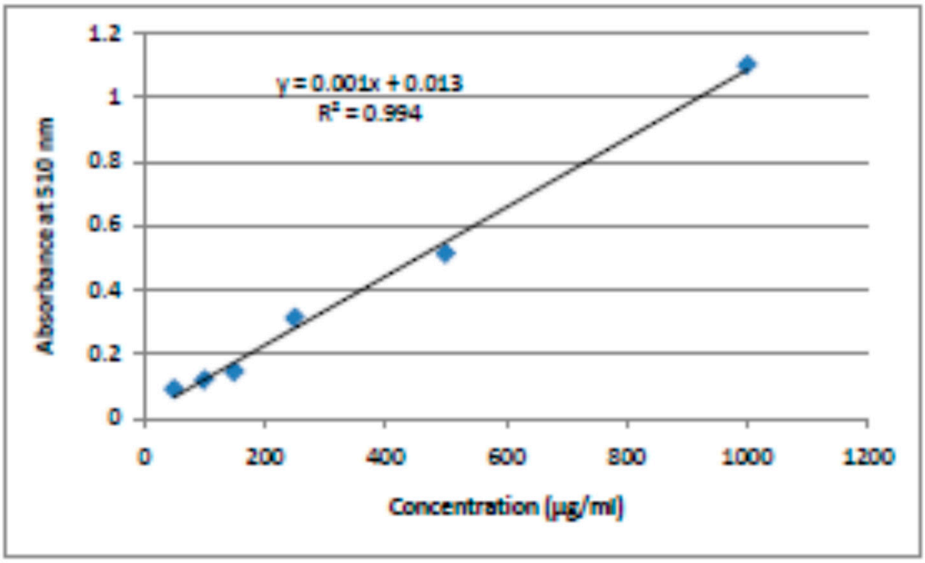

Figure 22S: Standard calibration curve of rutin used in the calculation of total flavonoid contents of *Z. coccineum*

D. *IN SILICO* BINDINGS:

Table S1: 3D structures, binding domains, and energies of the major constituents of *Z. coccineum*.

Compound	Relative percent of abundance	ΔG (Kcal /mol) at 4URO	3D Structure and binding domains	ΔG (Kcal/mol at 3QX3	3D Structure and binding domains
Tiliroside	19.80	-6.46	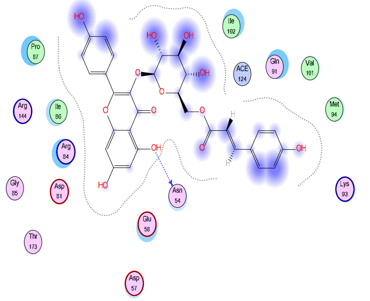	-6.63	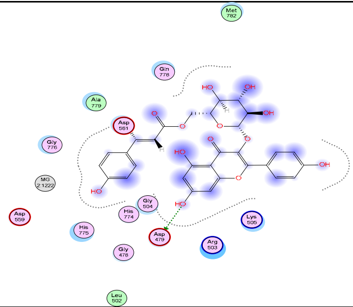
Zygophyloside-F	12.78	-6.06	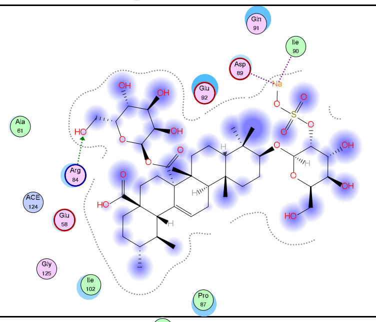	-6.22	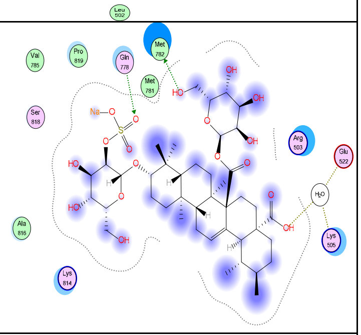
Isorhamnetin-3-O-glucoside	4.75%	-6.37	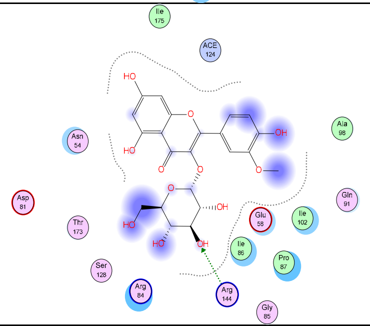	-5.75	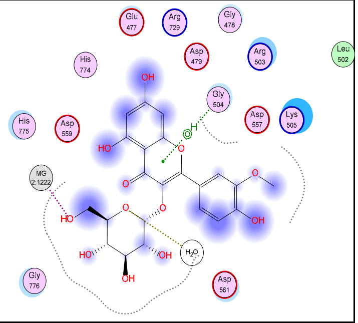
Kaempferol 3,7-di-O-α-L-rhamnoside	1.61	-6.47	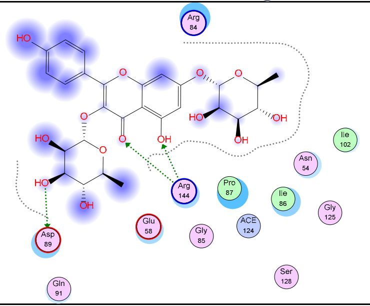	-5.65	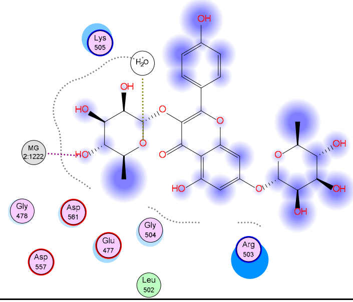
Luteolin	1.48	-5.19	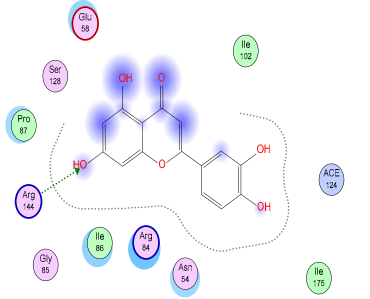	-5.08	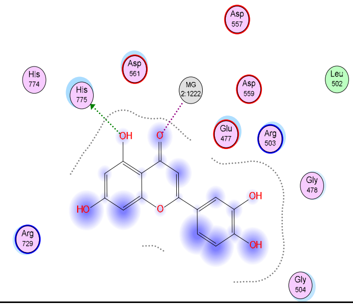

Spermine	0.94	-4.91	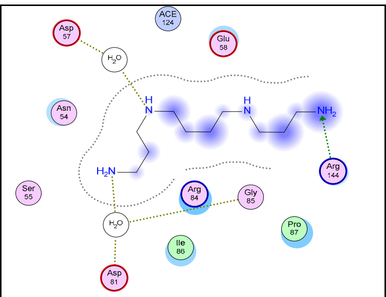	-5.10	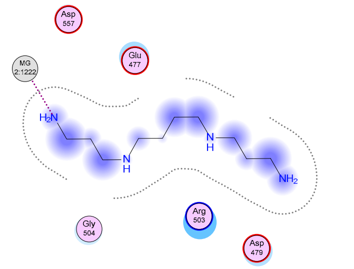
Gibberellin-A4	0.58	-3.81	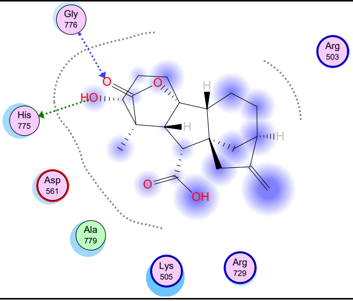	-4.23	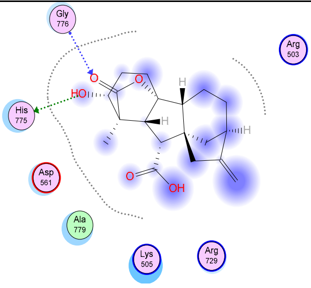
3-O-[β-D-Quinovo pyranosyl] quinovic acid-28-β-D-glucopyranosyl ester	3.31	-6.38	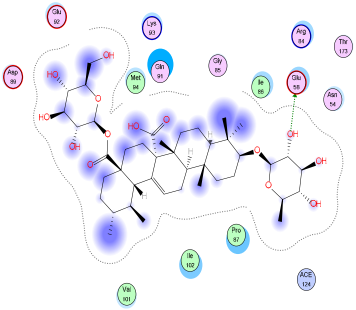	-6.23	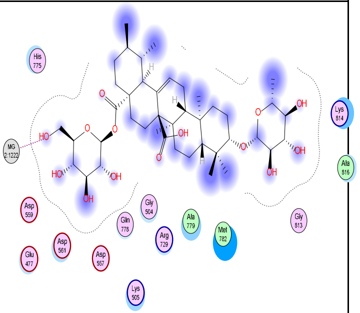
Caffeic acid	0.45	-4.6	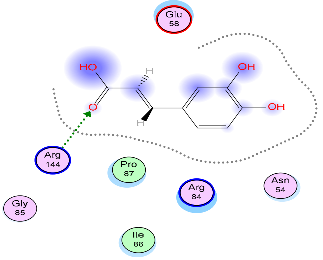	-4.6	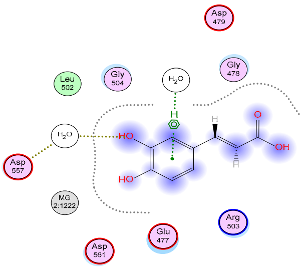
Etoposide				-5.94	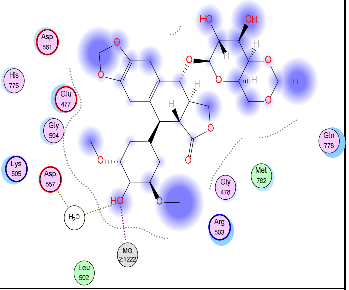
Novobiocin		-6.72	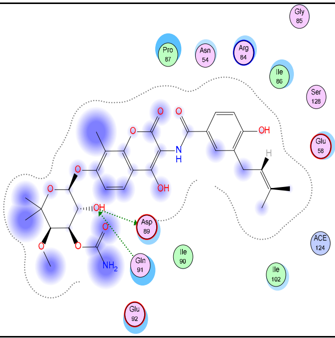		
